# Modeling the Effects of Protracted Cosmic Radiation in a Human Organ‐on‐Chip Platform

**DOI:** 10.1002/advs.202401415

**Published:** 2024-07-04

**Authors:** Daniel Naveed Tavakol, Trevor R. Nash, Youngbin Kim, Pamela L. Graney, Martin Liberman, Sharon Fleischer, Roberta I. Lock, Aaron O'Donnell, Leah Andrews, Derek Ning, Keith Yeager, Andrew Harken, Naresh Deoli, Sally A. Amundson, Guy Garty, Kam W. Leong, David J. Brenner, Gordana Vunjak‐Novakovic

**Affiliations:** ^1^ Department of Biomedical Engineering Columbia University New York NY 10032 USA; ^2^ Center for Radiological Research Columbia University New York NY 10032 USA; ^3^ Department of Biomedical Engineering Department of Medicine, and College of Dental Medicine Columbia University New York NY 10032 USA

**Keywords:** bone marrow, heart, human stem cells, liver, mars mission, organ‐on‐chip, radiation, tissue engineering

## Abstract

Galactic cosmic radiation (GCR) is one of the most serious risks posed to astronauts during missions to the Moon and Mars. Experimental models capable of recapitulating human physiology are critical to understanding the effects of radiation on human organs and developing radioprotective measures against space travel exposures. The effects of systemic radiation are studied using a multi‐organ‐on‐a‐chip (multi‐OoC) platform containing engineered tissue models of human bone marrow (site of hematopoiesis and acute radiation damage), cardiac muscle (site of chronic radiation damage) and liver (site of metabolism), linked by vascular circulation with an endothelial barrier separating individual tissue chambers from the vascular perfusate. Following protracted neutron radiation, the most damaging radiation component in deep space, a greater deviation of tissue function is observed as compared to the same cumulative dose delivered acutely. Further, by characterizing engineered bone marrow (eBM)‐derived immune cells in circulation, 58 unique genes specific to the effects of protracted neutron dosing are identified, as compared to acutely irradiated and healthy tissues. It propose that this bioengineered platform allows studies of human responses to extended radiation exposure in an “astronaut‐on‐a‐chip” model that can inform measures for mitigating cosmic radiation injury.

## Graphical abstract

In this work, the effects of galactic cosmic radiation are studied using a human multi‐organ‐on‐chip platform consisting of bone marrow (acute radiation damage), heart muscle (chronic radiation damage), and liver (metabolism). The tissues are matured in culture, linked by vascular circulation, and exposed to simulated cosmic radiation to assess their molecular, structural, and functional effects on human organs.

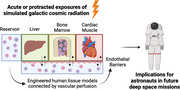

## 1 Introduction

Among the numerous “red risks” associated with space travel to the Moon and Mars, exposure to galactic cosmic radiation (GCR) has the highest potential to cause damage.^[^ ^1^, ^2^ ^]^ High linear energy transfer (LET) sources of cosmic radiation produce secondary neutrons upon contact with space vehicles, and these secondary neutrons are anticipated to be the most damaging ions in a future deep space mission. Accidental exposures to radiation have indicated significant correlations between high‐LET radiation levels and adverse effects on human health (i.e., cancer, heart disease).^[^ ^3^, ^4^ ^]^ Although the effects of high‐LET neutron exposures have been studied in animal models and human cells,^[^ ^5^, ^6^, ^7^, ^8^ ^]^ little is known about their effects on human organs.

Low‐dose protracted radiation exposures have been understudied in radiation biology, although it is hypothesized that prolonged cytotoxic stress may reduce cells’ ability to self‐repair, leading to accumulated DNA damage and epigenetic abnormalities.^[^ ^9^ ^]^ In animal studies, protracted radiation of heavy (56)Fe ions increased the incidence of acute myeloid leukemia (AML) via modifications of DNA methylation in hematopoietic stem and progenitor cells (HSPCs).^[^ ^10^ ^]^

Engineered human tissues have emerged over the last decade as models of human patho/physiology.^[^ ^11^, ^12^ ^]^ Tissues engineered from patient‐derived primary or induced pluripotent stem cells (iPSCs) using biomaterial scaffolds in conjunction with molecular and physical regulatory signals have been shown to recapitulate organ‐level functions, such as contractility of the heart, liver metabolism, and barrier function of the lung.^[^ ^12^, ^13^, ^14^ ^]^ These models enable individualized, patient‐specific studies of injury and disease.^[^ ^13^ ^]^ Various human tissues have been engineered, including heart muscle, bone marrow, liver, skin, brain, kidney, and lung.^[^ ^11^, ^12^ ^]^ Development of single‐organ models has enabled studies of multi‐organ interactions, by linking individual organ models into microphysiological platforms.^[^ ^15^, ^16^, ^17^ ^]^ Our group has demonstrated the use of such a platform for modeling interactions between multiple organs maintained in their respective environments (i.e., heart, liver, skin, bone) and connected by vascular circulation. To further mimic human physiology, an endothelial barrier separated the individual tissue compartments from vascular circulation while enabling selective transport of molecular species, extracellular vesicles, and cells—such as immune cells in response to tropic signals.^[^ ^18^, ^19^, ^20^ ^]^ These interactions are critical for communication within tissue models, as they modulate tissue responses to systemic stressors and the related production of cytokines and chemokines. Of particular interest are the progression of tissue injury and the return of injured tissues to homeostasis. In cases of drug studies, liver metabolism is critical for predicting pharmacokinetic and pharmacodynamic drug regimens for interacting organs.

Few studies to date have examined the effects of simulated cosmic radiation using engineered human tissues.^[^ ^21^, ^22^, ^23^ ^]^ We recently demonstrated the utility of individualized models of engineered cardiac muscle and bone marrow for studies of acute neutron radiation injury, which revealed potential markers of myeloid skewing in blood cells of the bone marrow and an early hypertrophic phenotype of the cardiac tissue.^[^ ^24^ ^]^

Here, we report the previously unknown effects of tissue exposure to simulated protracted cosmic radiation using a novel multi‐organ‐on‐a‐chip platform (multi‐OoC) with engineered human tissue models of bone marrow (eBM), cardiac muscle (eCT), and liver (eLiv). The tissue modules are linked by vascular circulation containing immune and blood cells, with a selectively permeable endothelial barrier separating the tissue compartments from circulating flow. In order to simulate extended low‐dose radiation exposure during a mission to Mars, we delivered the same total dose of high‐LET neutrons either acutely (high dose rate: a single dose of 0.5 Gy) or over 2 weeks (low dose rate: ≈0.04 Gy per day). Our model system is individualized and has allows potential for parsing out data between unique astronauts. We now begin to elucidate unique biomarkers associated with protracted radiation regimens, those of which have not yet been conducted in humanized settings in vitro. We characterized the functional and structural phenotypes of engineered tissues and the circulating blood and immune cells produced by the engineered bone marrow. By modeling human physiological responses to protracted space radiation, we seek to help develop radioprotective measures against damage anticipated during deep space travel.

## 2 Results

### 2.1 Multi‐OoC Platform with Heart, Bone Marrow, and Liver Tissues Linked by Vascular Flow

**Figure 1:**
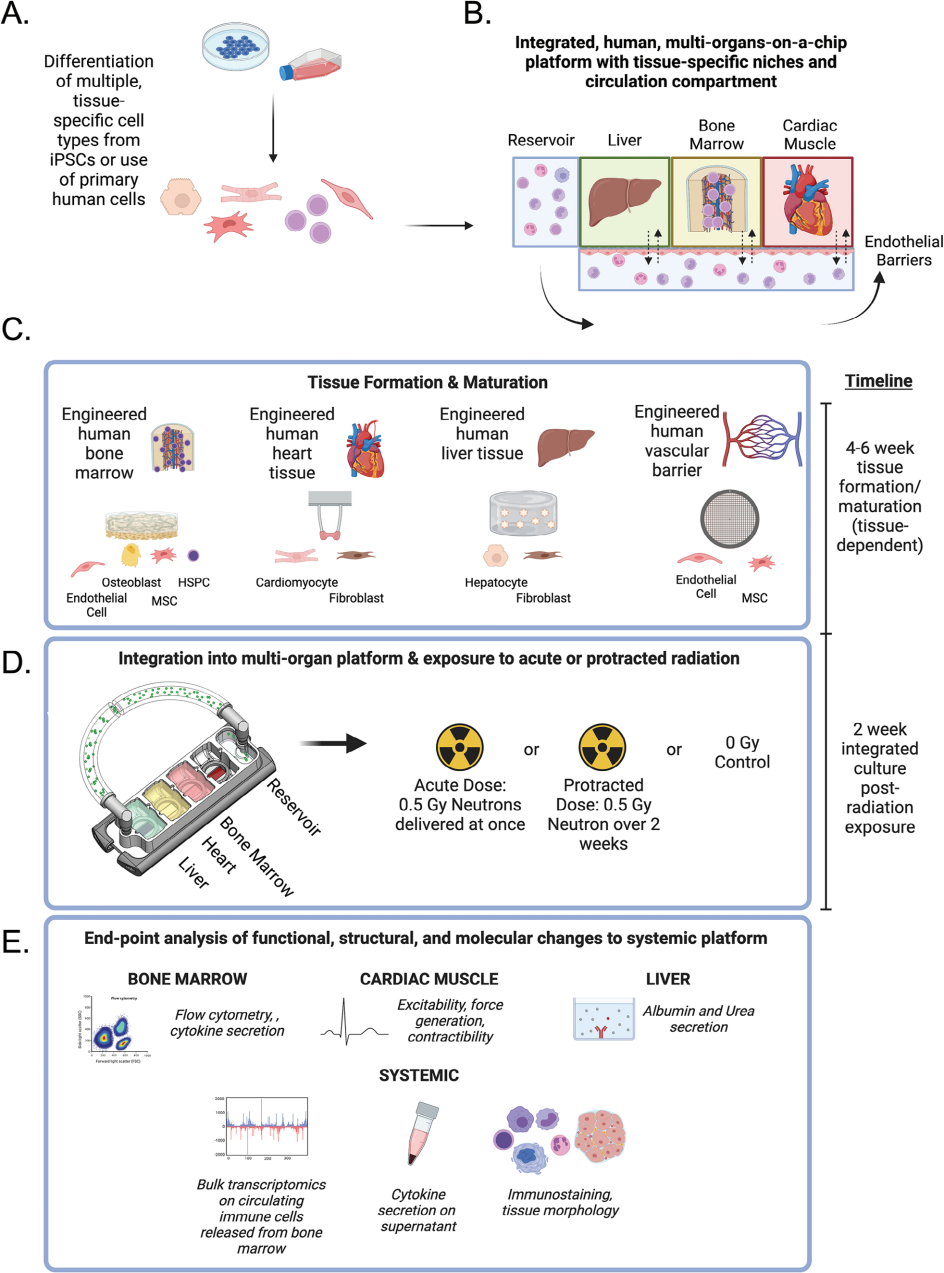
Overall approach. A) Differentiation of human iPSCs into tissue‐specific cells or sourcing of primary cells. B) Integrated, multi‐OoC platform with individual human tissue compartments, vascular flow, and endothelial barriers separating tissues from the flow.^[^ ^19^ ^]^ C) Individual tissue formation and maturation over a period of 4–6 weeks, followed by D) integration into the multi‐OoC platforms and exposure to an acute or protracted dose of 0.5 Gy of neutrons over two weeks at Columbia's Radiological Research Facility at Nevis Laboratories (see Figure S1 and Methods). E) End‐point assays performed to characterize the functional, structural, and molecular tissue phenotypes.

Our goal was to demonstrate the utility of a bioengineered, patient‐specific, multi‐OoC platform for studying doses and types of ionizing radiation relevant to space travel. We engineered human tissue models of bone marrow, cardiac muscle, and liver, each in its optimal environment, with selectively permeable vascular barriers separating the individual tissues from the circulating flow.^[^ ^19^, ^24^, ^25^ ^]^ (**Figure** **1**; Figures S2 and S3). The key scientific advance of this approach is that it allows the establishment and long‐term maintenance of the individual tissue phenotypes, along with their in vivo‐like communication via vascular flow containing immune cells generated by bone marrow.

To simulate the effects of GCR, we applied its most damaging component—high‐LET neutron radiation. We delivered either an acute dose of 0.5 Gy of neutrons (with ≈20% concomitant photons) or a protracted dose of ≈0.04 Gy of neutrons per day for a cumulative dose of 0.5 Gy over two weeks.^[^ ^8^ ^]^ The protracted regime was designed to more closely mimic continuous radiation exposure during spaceflight. We ended the experiment 24 h after the final dose of protracted exposure, providing each condition analyzed (control multi‐OoCs, acute 0.5 Gy‐exposed multi‐OoCs, and protracted 0.5 Gy‐exposed multi‐OoCs) with equal duration of culture after the initial radiation exposure (2 weeks after the first exposure for either acute or protracted). To characterize the responses to acute and protracted radiation, tissues were analyzed for morphological and functional changes, and culture media were analyzed for inflammatory cytokines and circulating immune cells (Figure 1).

### 2.2 Effects of Acute and Protracted Neutron Radiation on Hematopoiesis in Engineered Bone Marrow (eBM)

**Figure 2:**
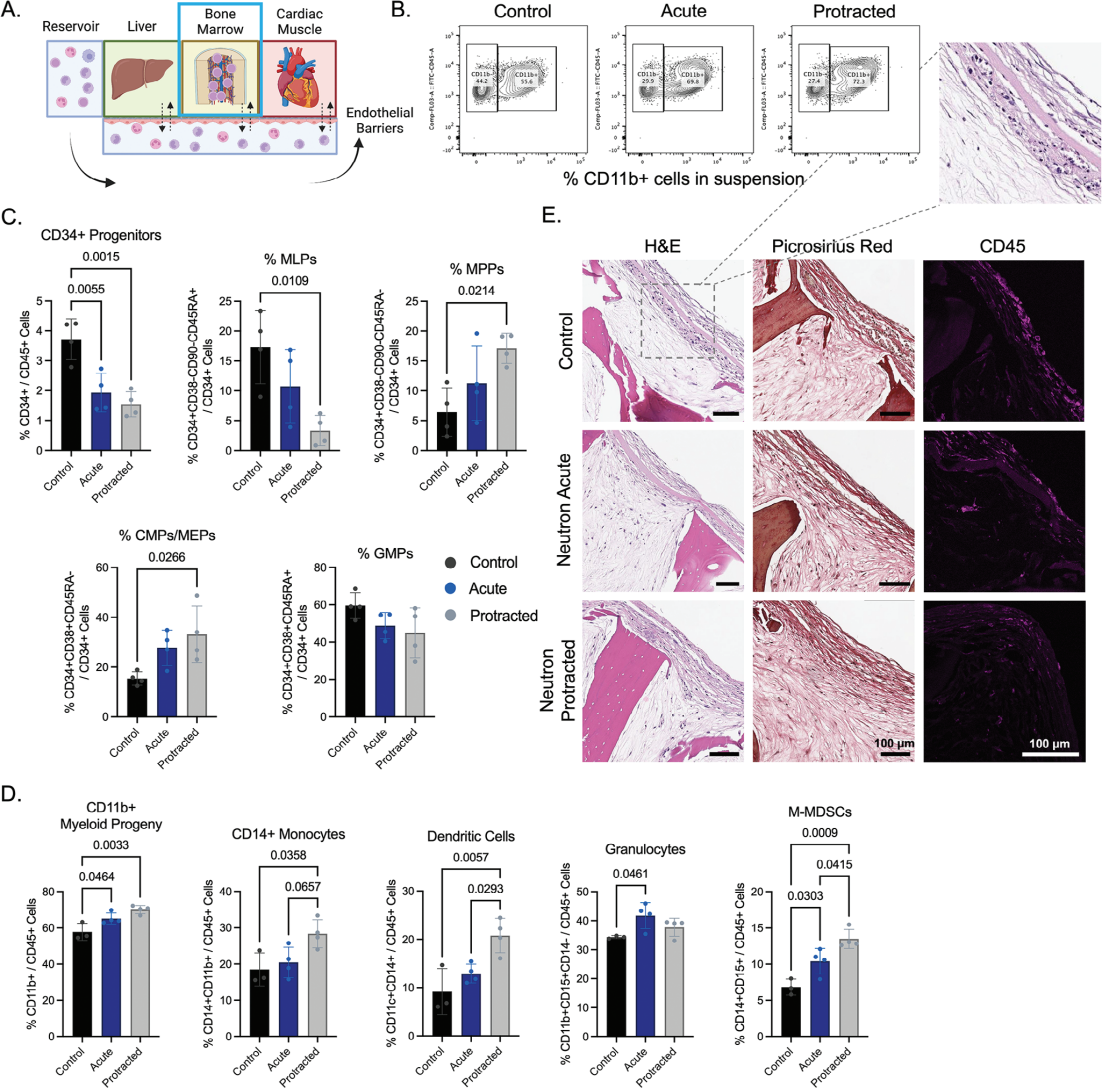
Functional and structural changes in eBMs 14 days after exposure to neutron radiation. A) Schematic of the platform with eBM tissue being characterized. B) Representative flow cytometric gating for CD11b+ cells in the eBM tissue compartment. C) Analysis of hematopoietic progenitors (CD34+, CD34+CD38‐CD90‐CD45RA+ MLPs, CD34+CD38‐CD90‐CD45RA‐ MPPs, CD34+CD38+ CD45RA‐ CMPs/MEPs, and CD34+CD38+ CD45RA+ GMPs) in multi‐OoC after exposures to acute (blue) or protracted (grey) neutrons, in comparison to healthy controls (black). D) Characterization of myeloid cells in eBM compartment (CD11b+ myeloid progeny, CD14+ monocytes, CD11c+CD14+ dendritic cells, CD11b+CD15+CD14‐ granulocytes, and CD11b+CD14+CD15+ M‐MDSCs). Significance was determined by one‐way ANOVA with Tukey's multiple comparisons test (*p*‐values not shown on plots are *p* > 0.1). E) Histological staining of eBMs using H&E, Picrosirius Red, and immunostaining for CD45.

At two weeks post‐tissue exposure to acute radiation or the first dose of protracted radiation, we characterized the structural and functional changes to the eBM (**Figure** **2**A). Flow cytometric analysis of the cells released from the eBM compartment revealed shifts in the scatters of CD45+ cells and increases in CD11b+ myeloid cells (Figure 2B; Figures S4 and S5).

Quantified percentages of CD34+ cells in the eBM showed a significant (p<0.01) decrease in the prevalence of hematopoietic progenitors in both acute and protracted radiation groups. This effect was even more pronounced in multi‐lymphoid progenitors (MLPs), which showed significant (p<0.05) reductions only in protracted and not acute radiation groups (Figure 2C).

In contrast, there were trending increases in the numbers of common myeloid and myeloerythroid progenitors (CMPs/MEPs) and multipotent progenitors (MPPs) in both irradiated groups, with only the protracted radiation group showing statistically significant increases (*p* < 0.05) in these progenitor subtypes. There were no significant changes to the percentages of granulocyte–monocyte progenitors (GMPs) (Figure 2C).

Similarly, there was a significant increase in the percentage of suspended CD11b+ cells in the eBM compartment in both groups, with a larger increase in the protracted than the acute group (Figure 2D). This effect was mirrored in multiple myeloid cell subtypes, including CD14+ monocytes, CD11c+CD14+ dendritic cells, and a putative population of CD14+CD15+ monocytic myeloid‐derived suppressor cells (M‐MDSCs). Notably, significant differences between the acute and protracted groups were only seen in dendritic cells (*p*<0.05), M‐MDSC (*p*<0.05), and non‐classical monocytes (p<0.05), although almost all cell populations demonstrated a trend of increase as compared to healthy controls (Figures 2C,D and S5A). Interestingly, using a generalized criterion for granulocytes (CD11b+CD15+CD14‐), we saw a significant increase in these cells two weeks post‐radiation only in the acutely irradiated eBMs (Figure 2D).

The increases in myeloid surface antigens and decreases in progenitor antigens were consistent with the trends in median fluorescent intensity (MFI) of CD45, CD14, and CD34 cells (Figure S5A). The cells adherent within eBM tissues showed significant decreases in CD34+ cell fractions and increases in CD11b+ cell fractions. There were no significant changes to the numbers of CD14+ monocytes, CD11c+CD14+ dendritic cells, and CD11b+CD15+CD14‐ granulocytes, indicating a radiation‐protective role of the niche (Figure S5B).

Histological analysis of eBM tissues stained with hematoxylin and eosin (H&E) and Picrosirius Red did not show changes between radiation conditions, except for decreased density of blood cells in the surface regions of the eBM tissues in irradiated groups (Figure 2E). Immunofluorescent staining confirmed a decrease in CD45+ blood cells in eBMs following protracted radiation, as compared to acutely irradiated and healthy control tissues (Figure 2F). These data are consistent with the flow cytometric analysis of eBMs in Figure 2B–D.

### 2.3 Effects of Acute and Protracted Neutron Radiation on eCT Function

**Figure 3:**
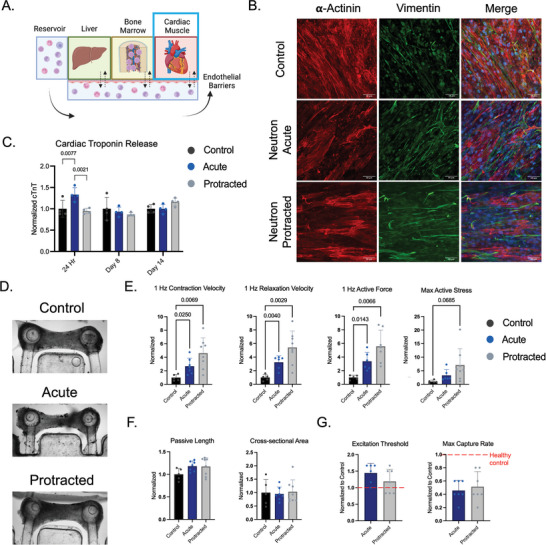
Functional and structural changes to eCTs 14 days after exposure to neutron radiation. A) Schematic of the platform with eCT tissue being characterized. B) Histological staining of eCTs using a canonical marker for cardiomyocytes (a‐actinin) and fibroblasts (vimentin). C) Cardiac troponin T (cTnT) concentration in the supernatant of eCT compartments over the duration of the experiment, normalized to control groups, with significance shown by two‐way ANOVA with multiple comparisons. D) Representative bright field image of eCTs at endpoint. E–G) Characterization of contractile function E), tissue morphology at rest F) at the 14‐day endpoint. Each tissue is normalized to itself at baseline and then normalized to the average value of the control group. G) Characterization of excitability of cardiac muscle tissues, normalized to the average value of the control group. Statistical significance was determined by one‐way ANOVA with Welch's correction and corrections for multiple comparisons (C,E,F) or by the Student's *t*‐test (G) (*p*‐values not shown on plots are *p* > 0.1).

eCTs were similarly analyzed for their structural and functional changes in response to acute or protracted regimens of radiation (**Figure** **3**A). To integrate the eCTs into the multi‐OOC platform, we incorporated the previously established electrical stimulation regimens.^[^ ^25^ ^]^ (Figure S6). Whole‐mounted eCTs showed visible changes in the presence of canonical markers of cardiomyocytes (*α*‐actinin) and fibroblasts (vimentin), with an increase in the number of vimentin+ and decrease in *α*‐actinin+ cells in both irradiated groups compared to healthy controls (Figure 3B). The concentration of cardiac Troponin T (cTnT) also increased in the acute‐irradiated eCTs 24 h after radiation exposure, as compared to both healthy controls (p<0.05) and protracted eCT groups (*p* < 0.01) (Figure 3C). This effect was neutralized one and two weeks after exposure, with no significant increases in cTnT secretion at any time point by eCTs exposed to protracted radiation.

Using live cell microscopy, we were able to characterize the changes in the functional capacity of eCTs in response to radiation at the 14‐day endpoint (Figure 3D). For protracted exposures, eCTs exhibited increases in maximum contraction velocity (*p* = 0.0069), relaxation velocity (p = 0.0029), active force (*p* = 0.0066), and active stress (*p* = 0.069) following neutron radiation (Figure 3E). There were nonsignificant changes in passive length (*p* = 0.22) and no changes to cross‐sectional area in protracted irradiated groups (Figure 3F). Excitation thresholds of irradiated eCTs were higher than the controls, while maximum capture rates were lower, though there were no significant differences in excitability or maximum capture rates between the two eCT groups exposed to radiation (Figure 3G). Taken together, these data indicate that there were significant changes to eCT function after radiation, especially in cases of protracted exposures.

### 2.4 Changes to eLiv Function Following Exposure to Acute and Protracted Radiation

**Figure 4:**
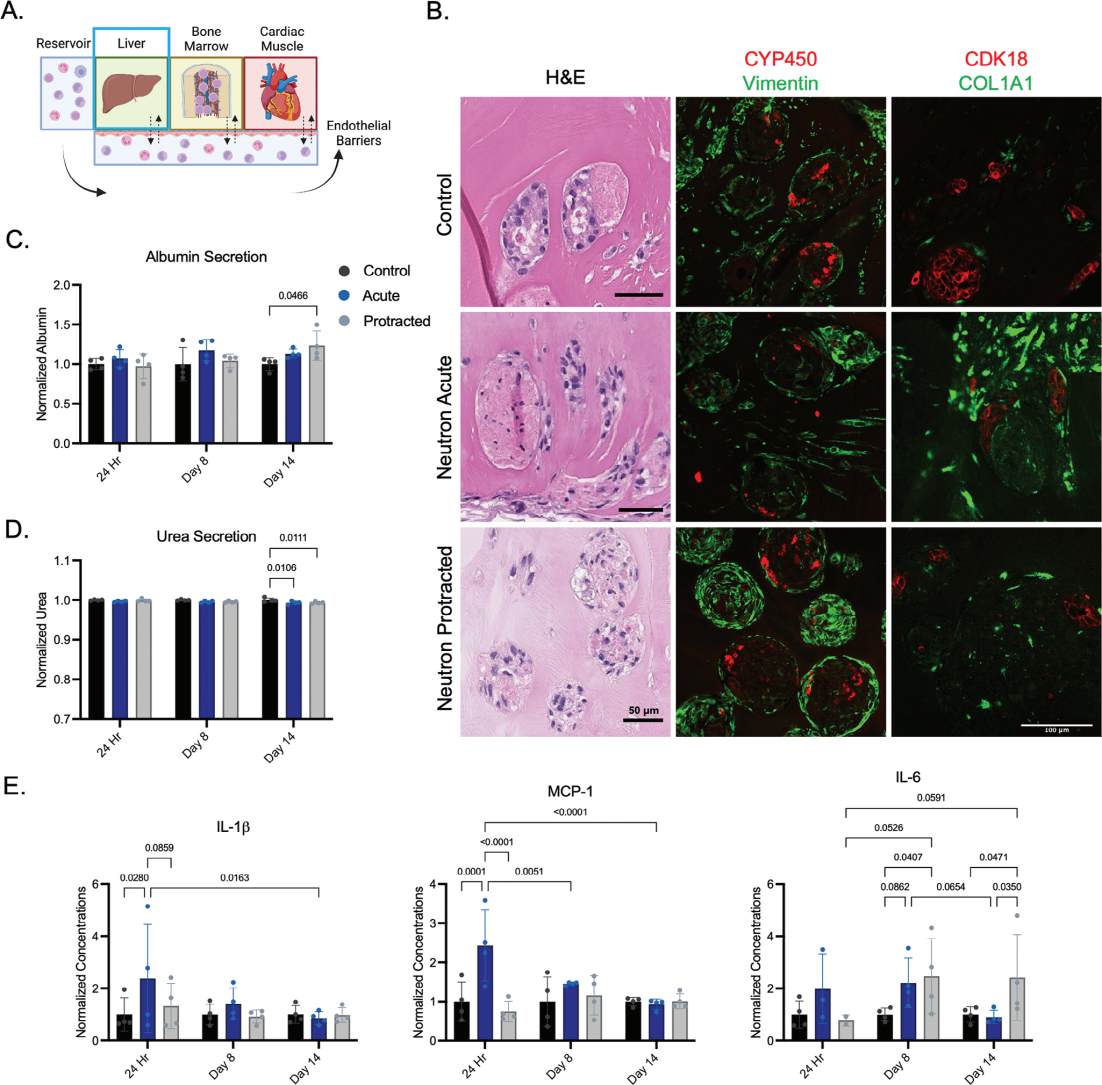
Functional and structural changes to eLivs after neutron exposures. A) Schematic of the platform with eLiv tissue being characterized. B) Histological staining of eLivs using canonical markers for hepatocytes (CYP450, CK18), fibroblasts (vimentin), and matrix deposition (COL1A1). C) Albumin and D) urea secretion in eLiv culture supernatant, normalized to the untreated controls. E) Cytokine secretion by eLivs 24 h, 1 week, and 2 weeks post initial radiation exposure, normalized to the untreated controls. Significance was noted by two‐way ANOVAs with multiple comparisons.

eLivs were analyzed for their secretion of well‐established indicators of hepatocyte function, and for morphological changes in response to radiation injury (**Figure** **4**A). Structurally, there were no significant changes observed in the H&E staining of liver aggregates within the hydrogel. Throughout the culture period, expression of cytochrome P450 (CYP450; a marker of metabolism) and cytokeratin 18 (CK18; a marker of hepatocytes) was detected in control, acute, and protracted irradiated groups. Notably, there was greater COL1A1 secretion in acutely irradiated eLivs, as compared to the other two groups (Figure 4B).

There were also no significant changes in albumin content in supernatant from the eLiv compartment immediately after radiation exposures, apart from an increase among protracted eLiv tissues at day 14 (Figure 4C). Similarly, after two weeks, there was a slight significant reduction in urea production by eLivs exposed to either acute or protracted radiation (Figure 4D). We identified immediate increases in IL‐1b, MCP‐1, and IL‐6, in eLiv supernatant 24 h post‐irradiation (Figure 4E). These cytokines were restored to control levels at one‐ and two‐week timepoints, with the exception of IL‐6, which remained elevated in the protracted radiation group (Figure 4E).

### 2.5 Changes to Circulating Immune Cells Following Exposure to Acute and Protracted Radiation

**Figure 5:**
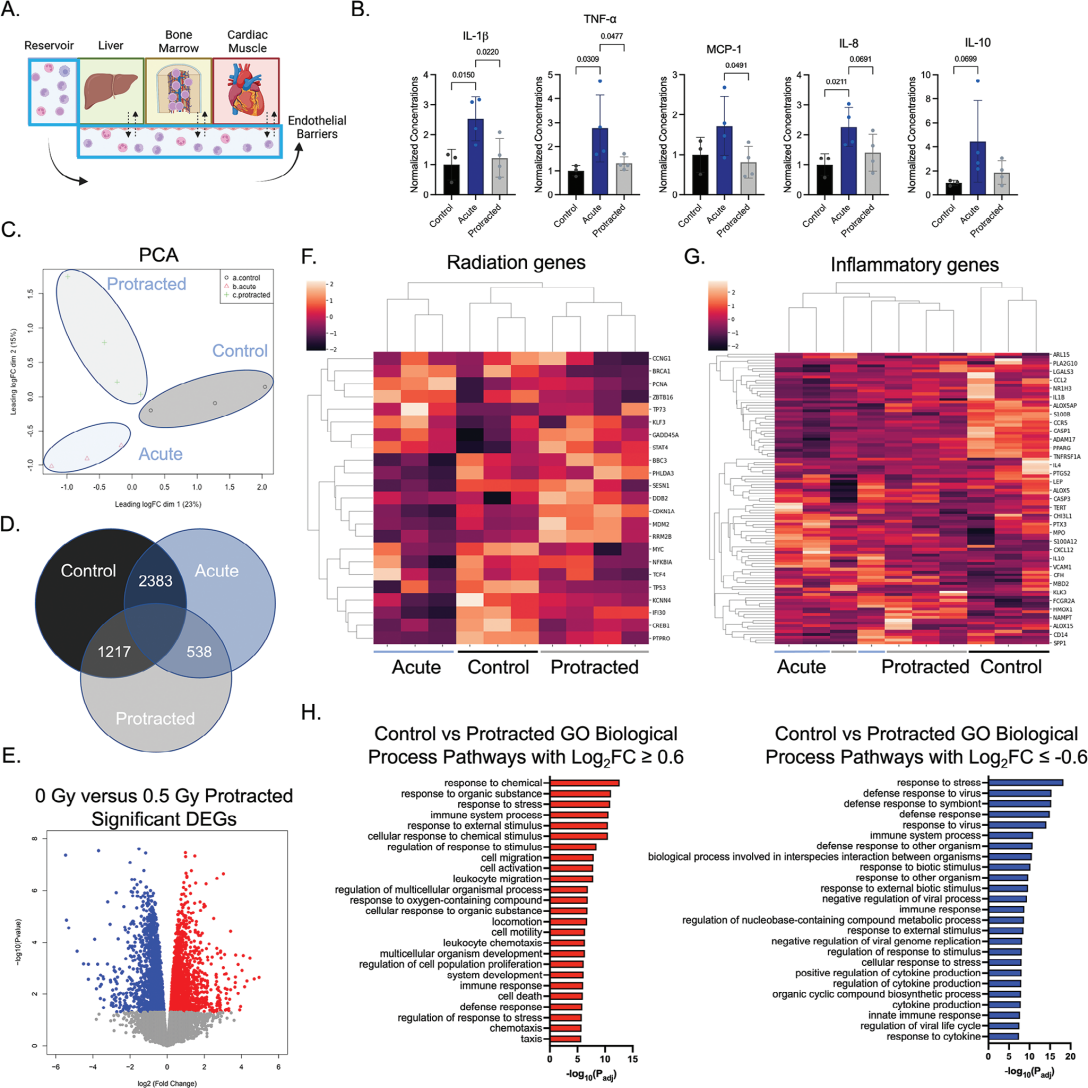
Transcriptomic changes in eBM‐produced circulating immune cells in response to protracted radiation exposure. A) Schematic of the platform with circulating cells being characterized. B) Secreted inflammatory cytokines in the circulatory compartment 24 h post‐irradiation. Statistical significance was performed via one‐way ANOVA without correction for multiple comparisons. C) Principal component analysis (PCA) of control, acute, and protracted circulatory cells gene expression. D) Differentially expressed genes (DEGs) in immune cells in the control versus acute group, control versus protracted group, and acute versus protracted group. E) Volcano plot of significant DEGs in protracted group as compared to healthy controls. F, G) Heatmaps of common radiation damage F) and inflammatory genes (G). H) Gene ontology (GO) pathway analysis of biological processes for upregulated (left) and downregulated (right) processes for protracted versus control groups.

One of the unique aspects of our model system is the ability of the eBM to produce and release immune cells into circulation, allowing their interactions with other tissues (**Figure** **5**A). We measured supernatant levels of inflammatory cytokines in the vascular perfusate 24 h after acute radiation and the first protracted radiation dose, which showed significant increases in IL‐1b (*p* < 0.05) and IL‐8 (*p* < 0.05), with nonsignificant increases in TNF‐a, MCP‐1, and IL‐10, indicating pan‐inflammatory responses of circulating immune cells and the endothelial barriers in the acute samples (Figure 5B).

**Figure 6:**
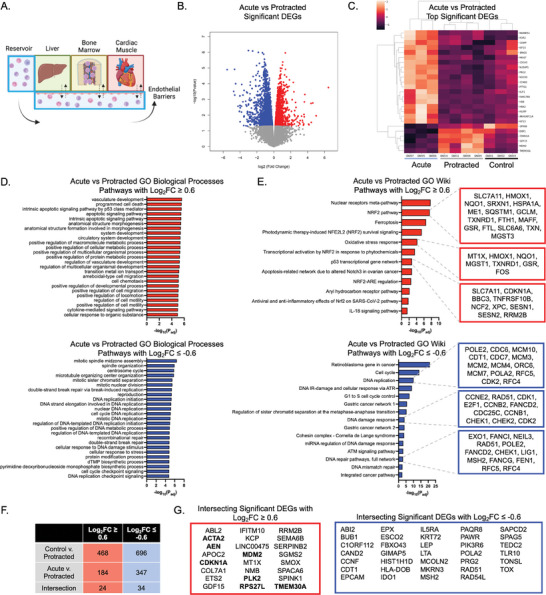
Transcriptomic changes in circulating immune cells following protracted radiation exposure. A) Schematic of the platform with circulating cells being characterized. B) Volcano plot of significant DEGs in the protracted radiation group as compared to the acute radiation group. C) Heatmap of top DEGs. D) GO pathway analysis of upregulated (top) and downregulated (bottom) biological processes following exposure to protracted as compared to acute radiation. E) GO analysis of Wiki Pathways resulting in upregulated (top) and downregulated (bottom) genes following protracted versus acute radiation, with identification of target genes enriched in each pathway. F) Identification of genes specifically responding to protracted radiation exposure. G) List of genes with increased (left, red) and decreased (right, blue) fold changes following exposure to protracted radiation, as compared to either acute radiation or healthy controls. Known radiation response genes are shown in bold.

At the two‐week endpoint, we isolated RNA from the circulating cells and analyzed their transcriptomic responses to the two radiation regimens by bulk RNA sequencing (Figures 5 and **6**; Figures S7 and S8). To validate the use of bulk RNA sequencing, we used CIBERSORTx.^[^ ^26^ ^]^ to identify the presence of monocytes, macrophages, dendritic cells, and granulocytes in all groups (Figure S7). Clustered tissue samples at each condition that were seen by principal component analysis (Figure 5C) revealed 2383 significant differentially expressed genes (DEGs) (FDR<0.05) identified between control and acute radiation OoCs, 1217 DEGs between control and protracted radiation OoCs, and 538 DEGs between acute and protracted radiation OoCs (Figure 5D,E).

We also noticed canonical expression changes associated with radiation damage, including decreases in *MYC* and increases in *PHLDA3*, *CDKN1A*, and *MDM2*, among others, with further changes between acute‐ and protracted‐exposed cells (Figure 5F). Inflammatory genes also varied, though all radiation‐exposed circulating cells clustered together regardless of the dosing regimen, indicating that a radiation‐specific response may supersede the timing of doses (Figure 5G).

As protracted radiation exposure may simulate more realistically the radiation during space travel than acute exposure, we rooted our analysis in comparisons between these tissues and the acutely irradiated and healthy controls. Among the 1217 DEGs between the protracted radiation and control multi‐OoCs (Figure 5D), we identified all genes showing Log_2_ Fold Changes (L_2_FC) ≥ 0.6 or L_2_FC ≤ −0.6 (Tables S1–S3 for the top 40 genes per comparison). We then subjected significant DEGs with either positive or negative L_2_FCs to gene ontology (GO) analysis to assess the upregulation of biological pathways associated with “response to stress,” “leukocyte migration,” “cell death,” and all pathways associated with “acute cytotoxic immune injury” (Figure 5H). In downregulated pathways, we found “immune system processes,” “regulation of cytokine production,” and “innate immune response,” indicating potential myeloid cell dysfunction in the circulatory compartment (Figure 5H). Notably, radiation and DNA damage response genes *EDA2R* and *MSX1* were identified within the top five genes upregulated in the protracted radiation groups (Table S2).

To identify genes unique to protracted exposures, we assessed differential gene expression in circulating immune cells relative to the acute condition (Figure 6A), resulting in 184 upregulated genes and 347 downregulated genes in the protracted group (Figure 6B). Among the top significant DEGs, acute radiation caused increases in canonical marker genes for myeloerythroid cells, including *HBB* and *HBA2*, and proliferating cells including *MKI67* and *CDCA3*. Protracted radiation in turn caused significant increases in radiation damage biomarkers including *TMEM30A*, *MDM2*, and *CDKN1A* (Figure 6C).

When analyzing genes with a cutoff of L_2_FC > 0.6, we identified upregulated GO biological process pathways including “intrinsic apoptotic signaling pathway by p53 class mediator,” “circulatory system development,” and “positive regulation of cell migration” (Figure 6D). Similarly, analyzing downregulated genes with L2FC < −0.6, revealed pathways associated with acute exposures, including “double‐strand break repair via break‐induced replication,” “cell cycle DNA replication,” and “cell cycle checkpoint signaling.” Most pathways were associated with cell cycle process responses to double‐stranded DNA damage (Figure 6D).

Using the Wiki Pathways database, we identified upregulated pathways in protracted radiation groups that included “NRF2 pathway,” “ferroptosis,” “oxidative stress response,” and “p53 transcriptional gene network” (Figure 6E). Genes associated with these pathways include common genetic biomarkers of p53 signaling and tumor suppressors (*SLC7A11, CDKN1A, TNFRSF10B*), as well as genes associated with oxidative stress response (*HMOX1, NQO1, FOS*) (Figure 6E). Downregulated genes in protracted radiation groups revealed Wiki Pathways including “DNA replication,” “DNA damage response,” and “DNA repair pathways,” implicating cell cycle checkpoint genes such as *RAD51, CHEK1, CHEK2*, and *CDK2* (Figure 6E).

In addition, we sought to identify genes that were upregulated or downregulated in protracted radiation groups as compared to both the controls and the acutely irradiated multi‐OoCs that may indicate biomarkers of protracted radiation injury. We identified 24 upregulated and 34 downregulated genes (Figure 6F), including several significantly upregulated genes previously implicated in responses to radiation (*ACTA2*, *AEN*, *CDKN1A, MDM2, PLK2, RPS27L, TMEM30A*) (Figure 6G). We also identified a number of genes downregulated following protracted radiation, including those implicated in antigen presentation (i.e., myeloid markers) and DNA damage repair (i.e., cell cycle) (Figure 6G).

## 3 Discussion

We report the development of a human organ‐on‐a‐chip model for modeling the effects of cosmic radiation, with engineered tissues (bone marrow, heart, liver) maintained in individualized compartments connected via circulating flow and separated by endothelial barriers. Human cells were combined with tissue‐specific scaffolds, in addition to molecular and biomechanical cues, to engineer and mature micro‐sized human tissues to recapitulate organ‐level functions (production of immune cells by bone marrow, contractile function of the heart, production of albumin and urea by liver). The platform was exposed to high‐LET neutrons and used as a proxy for the most damaging component of GCR in deep space, secondary neutrons. Our goals were to identify tissue‐specific responses to protracted radiation at the levels and rates expected during long‐range space missions and to compare the effects of protracted and acute exposures with equivalent doses of radiation and to non‐irradiated controls. Ultimately, this study established a framework for investigating the effects of space radiation on human tissues and circulating immune cells.

Exposure to low‐dose protracted radiation is among the highest risks and largest unknowns associated with deep space travel. For over 100 years, studies of the effects of acute radiation on human health have informed the development of radioprotective agents and the design of protocols for radiation use in cancer therapy.^[^ ^9^ ^]^ Effects of protracted radiation representative of GCR on human tissues have been poorly characterized, primarily due to limitations of traditional tissue culture techniques. Observational studies of extended, low‐dose environmental radiation exposures have indicated increases in the incidence of leukemia and solid tumors, similar to the rates seen among atomic bomb survivors in Japan.^[^ ^3^, ^4^, ^27^ ^]^ Extended exposures to low doses of radiation also increase the risk of genetic abnormalities and oxidative stress that prevent healthy cells from repairing accumulated DNA damage and lead to health complications.^[^ ^9^, ^28^ ^]^

After acute radiation exposure, eBM tissues displayed decreased fractions of hematopoietic progenitors (CD34+ HSPCs) and increased fractions of myeloid‐biased progenitors (CMPs/MEPs) and progeny (CD11b+, CD14+) (Figure 2). In addition to decreases in the fractions of MLPs in irradiated groups, these data are in line with reports of myeloid skewing in blood progeny of astronauts returning from space.^[^ ^29^ ^]^ Among tissues exposed to protracted radiation, we noticed an even greater decrease in hematopoietic progenitors and a larger increase in myeloid skewing than in response to acute radiation. These effects suggest that extended exposure to low‐dose neutron radiation more effectively promoted myeloid differentiation, as a result of non‐transient injury of HSPCs in the marrow. In multi‐year studies in canines, daily doses of 0.3 to 26.3 cGy resulted in significant suppression of the total output of blood cells, with the incidence of aplastic anemia and myeloproliferative neoplasms appearing at doses between 3.75 and 7 cGy day^−1^, well within the range of our experimental dose of ≈4 cGy day^−1^. Although hematopoietic damage was also observed following acute radiation exposure, protracted exposures at lower radiation doses resulted in further skewing of hematopoietic progeny toward myeloproliferative neoplasms or leukemias.^[^ ^30^, ^31^ ^]^ Cumulative DNA damage and the harbored environment of inflammation and oxidative stress in the marrow niche may work in concert to induce malignant hematopoiesis.^[^ ^32^, ^33^ ^]^ We believe this is also the case in our study, allowing for the accumulated DNA damage in the protracted irradiation regimens to promote accelerated differentiation and increased apoptosis of CD34+ progenitors.

Our data show significant increases in contraction velocity, relaxation velocity, and force generation in eCTs subjected to protracted radiation, with greater deviations from the controls in protracted tissues as compared to acute 0.5 Gy neutron‐exposed tissues (Figure 3E). These effects, in conjunction with increases in excitation threshold and decreases in maximum capture rate in all irradiated eCTs, are consistent with our previously observed neutron‐specific increases in eCT force generation.^[^ ^2^, ^24^ ^]^ In particular, increases in functional force generation are potentially predictive of an early hypertrophic cardiac phenotype, as seen in mouse models of low‐dose radiation damage, including matrix remodeling, changes to contractile function, and ventricular wall stiffening.^[^ ^34^, ^35^, ^36^ ^]^

In comparison, the functional changes in eLiv tissues only appeared after two weeks of exposure to either acute or protracted radiation. However, we noticed increased inflammatory cytokine secretion in eLivs 24 h after acute neutron radiation, as well as increased collagen 1 deposition in histological sections of these same tissues at two weeks post‐injury (Figure 4). Hepatocytes have been shown to be relatively radioresistant, though high doses of ionizing radiation have resulted in increased alanine transaminase (ALT) or aspartate aminotransferase (AST) levels in patient serum.^[^ ^37^ ^]^ It has been suggested that these disparities in hepatocyte function and liver health could be related to the non‐parenchymal fibroblasts increasing paracrine inflammatory signals in the liver.^[^ ^38^, ^39^ ^]^ This effect is similarly seen in our model, potentially due to the presence of fibroblasts in co‐culture with hepatocytes.

Transcriptomic data for circulating cells in this study suggest that protracted radiation upregulated the pathways related to apoptosis, stress response, and migration, and suppressed immune functions such as host‐defense response and cytokine production (Figure 5). Additionally, protracted radiation resulted in similar upregulation of p53‐mediated cell injury and oxidative stress pathways as seen in acutely irradiated tissues (Figure 6). Circulating immune cells in the acute radiation group were upregulated in pathways associated with a post‐DNA damage response, including cell cycle and DNA replication, potentially indicating that these cells are past radiation‐induced damage expression and are instead proliferating to recuperate from cytotoxic injury. Most interestingly, a comparison of protracted radiation to healthy controls or acute radiation exposure revealed 24 unique genes upregulated following protracted radiation exposure, including many radiation response genes (*ACTA2, AEN, CDKN1A, MDM2, PLK2, RPS27L, TMEM30A*).^[^ ^40^ ^]^ These upregulated genes may serve as biomarkers of protracted radiation damage and as potential targets for radiation protection in long‐range deep space missions.

Data obtained from astronauts returning from the International Space Station have revealed a post‐flight inflammatory stress pattern.^[^ ^41^ ^]^ Exposure of peripheral human blood ex vivo to mixed neutron radiation resulted in immediate increases in expression levels of genes related to DNA damage and p53 pathways.^[^ ^42^ ^]^ These responses were dose‐dependent, indicating these radiation damage‐related genes as potential markers of neutron‐specific injury (i.e., *BAX, TNFRSF10B, AEN*). Notably, the dose interval of the cumulative radiation delivery was critical to observed proportional differences in cell survival and proliferation. In vitro, cultures of cancer cells treated over extended radiation intervals were more resilient than those treated over shorter and more frequent intervals.^[^ ^43^ ^]^ Although our study delivered protracted exposures once a day for two weeks, it is expected that around‐the‐clock radiation exposures, as envisioned in a long‐range mission to Mars, maybe even more damaging to human tissues.

Engineered human tissue models cultured within a systemic context thus enable assessment of multi‐tissue interactions in response to stressors. Several research groups, including ours, have developed platforms that allow multiple engineered tissue models (i.e., multi‐OoCs) to be connected for systemic modeling, and to include liver‐mediated drug metabolism.^[^ ^15^, ^17^, ^18^, ^19^, ^44^, ^45^, ^46^, ^47^ ^]^ In the platform described here, a critical living bone marrow compartment serves as the primary site of immune cell production and depot for recapitulating systemic inflammatory responses, enabling migration of immune cells into the circulatory compartment, which can interact with all tissue sites in the multi‐OoC model.

Although we demonstrate the feasibility of using a human multi‐OoC model for studying the effects of high‐LET acute and protracted exposures, there are several limitations of our work that may inform future studies.

First, we chose to include only a handful of tissue models: bone marrow as a target of acute radiation damage to an adult stem cell population, heart as a target of chronic radiation damage with limited regenerative capacity, liver as a site of metabolism, and the connecting vascular endothelial lining to separate the flow channel with circulating cells and tissue‐specific secreted factors from the tissue compartments. Clearly, there is a need to assess responses in other critical organ systems (such as the brain, kidney, skin, and lungs). The modular, open design of the platform allows for these extensions in future studies. By incorporating other tissue models, we may be able to better elucidate changes to other stem cell niches (i.e., lung and gut) and identify protective measures that span multiple stem cell populations. Critical regulators of cognitive function (i.e., brain) also tie into other “Red Risk” stressors identified by NASA of concern in deep space missions.

Second, our engineered models incorporate only a subset of cell types for recapitulating the tissue‐specific functions in vitro. There is more work ahead to elucidate the relationships between parenchymal cells and their microenvironments during radiation exposure.^[^ ^48^ ^]^

Third, we analyzed the protracted radiation exposure over a period of two weeks, which should be extended to longer time intervals (many months) and a variety of radiation doses.^[^ ^2^ ^]^ Longer‐term studies would enable the assessment of longer‐lasting changes to the tissues, indicating whether radiation biomarkers return to normal after removal of the radiation stressor (i.e., mimicking an astronaut returning to Earth).

These efforts are already ongoing in multiple laboratories, with the goal to extend the lifetime of organ‐on‐a‐chip systems to up to 6‐months. In addition, the facilities at the Brookhaven National Laboratories are hosting investigators to provide improved simulation of galactic cosmic radiation for studies in human organs‐on‐chip platforms. Although secondary neutrons are an effective proxy for simulated GCRs, future work should compare the varying radiation regimens to better mimic the space environment.

The present study is based on a long‐term mission during which all organs would be expected to accumulate significant radiation exposure. During actual space travel, however, individual organs could experience particle traversals to a greater extent than the remainder of the body. Future studies can be designed to investigate effects across organ systems using our organ‐on‐a‐chip model and a targeted beam to irradiate different components at different times, with different radiation doses, or with different particles.

Finally, future studies of engineered human tissues and OoC systems should utilize the advances in stem cell biology to evaluate the effects of biological diversity (sex, race, age) and identify factors that predict susceptibility to radiation damage.^[^ ^11^, ^12^, ^13^ ^]^ The current state of the art allows us to combine bioengineered tools with pre‐clinical models and clinical data to predict human body responses to deep space radiation exposure. As the operation of multi‐OoC platforms can be automated, we envision that these studies are proof of the feasibility of adapting such systems for long‐term durations of radiation exposure in space missions.

## 4 Conclusion

We studied the systemic effects of acute and protracted radiation using a multi‐organ platform with bioengineered human tissues—heart, liver, and bone marrow—in experiments designed to simulate the effects of high‐LET exposures encountered during space missions. Our tissue‐specific data suggest that protracted doses of high‐LET radiation result in larger changes in tissue functions as compared to a single acute dosage. By analyzing the transcriptomic profiles of engineered bone marrow‐derived immune cells that have reached the circulatory compartment, we were able to identify multiple genes of interest for the development of deep‐space radioprotective agents. We believe that this study is the first to demonstrate the feasibility of using bioengineered tissues for assessing the effects of cosmic radiation in an “astronaut‐on‐a‐chip” approach, providing a basis for developing radioprotective measures for mitigating long‐term radiation damage.

## 5 Experimental Section

### Study Design

The model system was a configuration of an all‐human modular organs‐on‐chip platform, with tissues linked to each other by vascular flow and an endothelial barrier separating the tissue and vascular compartments.^[^ ^19^ ^]^ To study the effects of acute and protracted radiation, three target organs were chosen: cardiac muscle (site of chronic radiation damage), bone marrow (site of acute radiation damage), and liver (site of metabolism), along with the vascular barrier. Each tissue was engineered and matured separately using previously established protocols,^[^ ^18^, ^19^, ^24^, ^25^ ^]^ evaluated, and quality controlled before being transferred into the platform for integrated culture and exposure to radiation. To mimic the conditions expected in deep space travel to Mars, a cumulative dose of 0.5 Gy neutron radiation was chosen for all tissues.^[^ ^49^ ^]^ As astronauts in Mars missions will not receive just one acute dose and will instead be exposed to radiation over the entire duration of their mission, the acute dose of 0.5 Gy was compared with the protracted dose of 0.5 Gy delivered over a 2‐week period at 0.04 Gy day^−1^. Throughout the study and in particular, at the 2‐week endpoint, the molecular, structural, and functional changes were characterized for all tissues and the circulating cells produced in the bone marrow (Figure 1).

### Platform Fabrication

The multi‐tissue platform.^[^ ^19^ ^]^ was configured by linking the individual tissue modules (bone marrow, liver, heart), each supplied with the optimal tissue‐specific medium, by vascular circulation. The vascular barrier, formed by seeding the endothelial cells and supporting mesenchymal cells onto a hydrophilic polyester (PETE) membrane, separated each compartment from the circulating flow (Figure 1B). The platform could fit inside a standard petri dish (Figure S1A,B) with dimensions roughly 26 × 75 mm, and a glass slide sealing off its bottom channel (Figure 1C). Pumps, tubing, connectors, and dishes were standard off‐the‐shelf components (Cole Parmer, Fischer Scientific). Pharmed tubing was used and cut to length to form a closed loop between the inlet and outlet to the platform and peristaltic pump.

The perfusion manifold was made from CNC‐machined polysulfone, with tissue chambers at the top side, and an open perfusion channel with vascular medium at the bottom side. The perfusion channel was fully enclosed by silicone O‐ring gaskets, a glass slide, and custom‐machined polycarbonate clamps that applied a compressive sealing force between the manifold, gasket, and glass (Figure S1). The endothelial barrier was established using a custom‐made insert fabricated by injection molding. A polypropylene core provided structural support for sealing the platform and the porous membrane. The polypropylene structure was inserted atop of a laser‐cut track‐etched polyester membrane with 8 µm pore size (Sterlitech). This assembly was over‐molded by injection molding with a thermoplastic elastomer (TPE) (Avient Versaflex, CL2242). The TPE served two functions: it anchored and sealed the PETE membrane to the polypropylene structure, and it provided a seal when installed into the fluidic manifold, separating the space between the vascular flow channel and the tissue‐specific reservoir. Tissues were fabricated and matured individually, as described below, and integrated into the multi‐OoC platform.

### Cardiac Differentiation of Human iPSCs

To visualize calcium handling in real‐time, WTC11‐GCaMP6f line of human iPSCs was used that contained a constitutively expressed GCaMP6f calcium‐responsive fluorescent protein inserted into a single allele of the AAVS1 safe harbor locus. The cells were obtained through a material transfer agreement from Dr. Conklin at Gladstone Institutes and differentiated into cardiomyocytes using a well‐established protocol.^[^ ^50^ ^]^ From day 10 to day 16, cells were cultured in RPMI‐no glucose medium (Life Technologies, 11879 020) supplemented with B27 (Thermo Fisher Scientific, 17504044) and 213 µg mL^−1^ ascorbic acid (Sigma–Aldrich, A445), to purify for iPSC‐CMs and eliminate contaminating mesodermal and endodermal cells. On day 17 cells were pretreated with rock‐inhibitor (y‐27632 dihydrochloride, 5 µm) for 4 h and dissociated by enzyme digestion with collagenase type II (95 U mL^−1^; Worthington, LS004176) and pancreatin (0.6 mg mL^−1^; Sigma–Aldrich, P7545) in dissociation buffer (Glucose (5.5 mm), CaCl2·2H20 (1.8 mm), KCl (5.36 mm), MgSO4·7H20 (0.81 mm), NaCl (0.1 m), NaHCO3 (0.44 mm), NaH2PO4 (0.9 mm)) on a shaker in a 37 °C incubator. Flow cytometry for cTnT+ (BD BioSciences, 565744) was performed to confirm the purity of iPSC‐CMs (>90% cTnT+).

### Engineering and Maturation of Cardiac Tissues

Cardiac tissues were made using our established protocol.^[^ ^25^ ^]^ Differentiated iPSC‐CMs were mixed with supporting primary human cardiac fibroblasts (NHCF‐V; Lonza, CC‐2904) in a 3:1 ratio and resuspended in RPMI‐B27 (RPMI 1640 basal medium supplemented with l‐ascorbic acid 2‐phosphate and B27). The cells were resuspended in human fibrinogen (Sigma, F3879) to a fibrinogen concentration of 5 mg mL^−1^ and cell concentration of 370,000 cells µL^−1^. Aliquots of 12 µL cells in fibrinogen and 3 µL of thrombin (2U mL^−1^) were added to each well and the mixture was left to polymerize at 37 °C for 15 min around the elastic pillars. Each well then received 400 µL of RPMI‐B27 with 213 µg mL^−1^ ascorbic acid, 10uM Rock inhibitor, and 5 mg mL^−1^ 6‐aminocaproic acid (Sigma, A7824). After 24 h, the cells were switched to a medium without ROCK inhibitor, and the medium was changed every other day. Starting from day 5 post‐tissue fabrication, 6‐aminocaporic acid was removed from the media.

Intensity training electrical stimulation was used to promote tissue maturation as previously described.^[^ ^51^ ^]^ Briefly, on day 7 following tissue preparation, tissues were electrically stimulated with biphasic electrical pulses (5 V cm^−1^, 2 ms duration, 2 Hz frequency). The stimulation frequency was gradually increased by 0.33 Hz per day until reaching 6 Hz (on day 19), and this frequency was maintained for two days. On day 21, the frequency was reduced to 2 Hz and maintained until day 28. RPMI‐B27 media was then replaced by media promoting metabolic maturation of iPSC‐CM (“maturation media”).^[^ ^52^ ^]^ Tissues were cultured at 2 Hz electrical stimulation for another seven days (until day 35), evaluated, and transferred into the platforms. Tissues remained in maturation media on‐chip under 2 Hz stimulation for the remainder of the study.

### Derivation of Bone Marrow Component Cells

iPSCs (WTC‐11 line) were derived as previously described using the STEMdiff Mesenchymal Progenitor Kit (Stem Cell Technologies, 05240), expanded, and frozen.^[^ ^24^ ^]^ Human umbilical vein endothelial cells (HUVECs; Lonza, C2519A) were expanded according to the manufacturer's instructions. CD34+ cord blood‐derived HSPCs (Stem Cell Technologies, 70008) were plated according to manufacturer's instructions in StemSpan SFEM II medium with 1% P/S (Stem Cell Technologies, 09655), StemSpan CD34+ expansion supplement (Stem Cell Technologies, 02691), and 1 µM of Pyrimido‐indole derivative UM729 for 4 days (Stem Cell Technologies, 72332).

### Engineering and Maturation of Human Bone Marrow Tissues

Fully decellularized trabecular bone blocks with preserved composition and microarchitecture of the bone matrix were fabricated into scaffolds measuring 4 × 8 × 1 mm, as previously described.^[^ ^18^, ^54^ ^]^ The resulting scaffolds were processed on an orbital shaker through a series of treatments: i) PBS with 0.1% EDTA (w/v) for 1 h at room temperature; ii) 10 mm tris, 0.1% EDTA (w/v) in DI water overnight at 4 °C; iii) 10 mm Tris, 0.5% sodium dodecyl sulfate (w/v) in DI water for 24 h at room temperature; iv) 100 DNase, 1 U mL^−1^ RNase, 10 mm Tris in DI water for 6 h at 37 °C. The scaffolds were lyophilized and freeze‐dried using a Labconco freezone lyophilizer (7740020) and controlled for porosity by selecting scaffolds that weighed 11–13 mg each. Scaffolds were immersed in 70% ethanol overnight and washed with DMEM overnight prior to use.

Each scaffold was seeded with 1.0 × 10^6^ iMSCs in 30 µL media (4.5 g L^−1^ DMEM supplemented with 10% (v/v) HyClone FBS, 1% penicillin/streptomycin, and 1 ng mL^−1^ of basic fibroblast growth factor, bFGF), according to established protocols.^[^ ^2^ ^]^ After 2 h of seeding, 1 mL of iMSC media was added and maintained for 72 h to allow for the iMSCs to attach and proliferate on the scaffold. Developing bone tissues were then switched for 4 weeks to osteogenic media consisting of DMEM with 1 g L^−1^ glucose, 100 nm dexamethasone (Sigma–Aldrich), 10 mm β‐glycerophosphate (Sigma–Aldrich), and 50 µM   l‐ascorbic acid‐2‐phosphate (Sigma–Aldrich). Media were changed every other day, allowing iMSCs to differentiate into osteoblasts.

Following osteogenic maturation, 150,000 HUVECs, an additional 150,000 iMSCs, and 40,,000 CB‐HSPCs were added within 16 µL of fibrin hydrogel (11 mg mL^−1^ fibrinogen, Sigma Aldrich, F3879; 33 U mL^−1^ thrombin, Sigma–Aldrich, T6884) to each bone tissue. eBMs were incubated at 37 °C in a humidified incubator at 5% CO_2_ for 20–25 min prior to being rehydrated with hematopoietic media. Tissues were fed with StemSpan SFEM II medium with 1% P/S (Stem Cell Technologies, 09655), 50 ng mL^−1^ stem cell factor (SCF), thrombopoietin (TPO), and FMS‐like tyrosine kinase 3 ligand (FLT‐3L) (Peprotech) with 1 µm UM729 and 33 mg mL^−1^ of the protease inhibitor aprotinin (Sigma–Aldrich, A3428) for the first 4 days. eBM tissues then underwent half media changes for two additional days with 50 ng mL^−1^ each of SCF, TPO, FLT‐3L in SFEM II medium prior to use in the multi‐OoC platform.

### Engineering of Liver Tissues

iPSC‐derived human hepatocytes were purchased from Cellular Dynamics (iHeps 2.0; CDI, R1027). Using an AggreWell plate with 400 µm microwells (Stem Cell Technologies, 34411), hepatocytes, and primary human dermal fibroblasts (Lonza) were mixed at a 1:1 ratio for the formation of liver spheroids, as previously described.^[^ ^19^, ^55^ ^]^ Spheroids were cultured in a hepatocyte culture medium (Lonza, CC‐3198) for two days in the AggreWell plates. Liver spheroids were then suspended in hepatocyte culture media and let them sink to the bottom of the tube over 20 min, and subsequently resuspended these spheroids in 84% fibrinogen (33 mg mL^−1^) and 16% thrombin (33 U mL^−1^). In each well, spheroids were suspended in 200 µL volume and let polymerize at 37 °C in a humidified incubator at 5% CO_2_ for 20 min, accounting for roughly 800,000 hepatocytes and 800,000 fibroblasts per tissue. 1 mL of culture medium containing 33 mg mL^−1^ of aprotinin (Sigma–Aldrich, A3428) was added to each well.

### Engineering and Maturation of the Endothelial Barrier

Endothelial mesh inserts made of polyester (PET) with 8 µm pore size were incubated in 70% ethanol overnight and washed three times in 1X PBS prior to use. Barriers were flipped upside down and incubated with 10 µg/mL human fibronectin (Corning, 356008) for 1 h prior to cell seeding, and washed twice with PBS. 400,000 HUVECs and 100,000 BM‐MSCs (ATCC, PCS‐500‐012) were seeded onto the inserts and incubated for 1.5–2 h in 50 µL of EGM‐2 media (Lonza, CC‐3162) at 37 °C in a humidified incubator at 5% CO_2_. The resulting barriers were incubated for 48 h in EGM‐2 media prior to integration into the multi‐OoC platform for maturation. Once introduced into the multi‐OoC platforms, peristaltic pump speed was accelerated every 12 h up to a final speed of 1.88 dynes cm^−2^ over the course of two days.

### Radiation Exposure

Neutron irradiations were performed at the Columbia University Radiological Research Accelerator Facility (RARAF), using an accelerator‐based neutron irradiator. This system was originally designed to mimic the neutron energy spectrum from an Improvised Nuclear Device,^[^ ^56^ ^]^ but has since been used to model the high LET component of GCR exposure.^[^ ^5^, ^53^ ^]^ To this end, a mixed beam of atomic and molecular ions of hydrogen and deuterium was accelerated to 5 MeV and used to bombard a thick beryllium target. The energy spectrum of neutrons emitted at the angle of 60° to the ion beam axis closely mimicked the Hiroshima spectrum at 1–1.5 km from the epicenter.^[^ ^8^ ^]^ During irradiation, the platforms were placed below and in front of the beryllium target, at an angle of 60° to the incident particle beam and a distance of 17.5 cm (Figure S1). Acute radiation exposures were performed with a total beam current of 28 µA, resulting in the prescribed dose of 0.5 Gy of Neutrons (with an additional 0.12 Gy of concomitant photons) delivered over 15–20 min. Protracted radiation exposures were conducted daily (12 daily fractions, six times a week over 2 weeks) using a beam current of 10–15 µA, and a dose of 0.042 Gy of neutrons and 0.01 Gy of concomitant photons) delivered over 3 min per fraction. Dosimetry was performed twice a week, as previously described,^[^ ^56^ ^]^ using a custom‐built Tissue Equivalent Proportional Counter (TEPC)^[^ ^7^ ^]^ which measured the total dose, and a compensated Geiger–Mueller dosimeter,^[^ ^57^ ^]^ which had a very low response to neutrons and thus measures only the photon component.

### Functional Analysis of Cardiac Tissues

Tissues were imaged in Brightfield to assess contractile force dynamics and in fluorescence to assess calcium handling using previously established methods.^[^ ^25^ ^]^ Force analysis of engineered tissues was performed by acquiring videos of tissue contraction under electrical stimulation at 1 Hz and analyzing the movement of the pillar heads. Displacement of pillars was measured using a previously described object tracking algorithm,^[^ ^25^ ^]^ and the amount of force exerted by the tissue at a given time was calculated using a measured Young's modulus of the pillar and the width of the tissue. The resulting force trace was analyzed to calculate contraction and relaxation velocities.

The excitation threshold (ET) and maximum capture rate (MCR) were determined based on the contractile response of the tissue at each stimulation setting, as described previously.^[^ ^53^ ^]^

For calcium imaging, tissues were subjected to 1 Hz stimulation. Videos were taken at 100 frames per second for 20 s and analyzed to extract functional metrics as previously described.^[^ ^25^ ^]^ In brief, a custom Python algorithm was used to compute pixel intensity in the tissue, which was then averaged to extract calcium transients over time. The resulting trace was further analyzed with SciPy, an open‐source Python library, to identify peaks and extract functional metrics (i.e., contraction/relaxation velocities, active force/stress, passive length).

### Collection of Cells for Flow Cytometry

At timed intervals, bone marrow tissues and the culture medium were harvested from the platform chambers for flow cytometric analyses. eBM tissues were washed twice with FACS buffer (2% FBS and 0.5 mm EDTA in 1× PBS) and digested in 10 mg mL^−1^ collagenase II (Worthington, LS004176) and 10 mg mL^−1^ Nattokinase (Japan Bio Science Lab) for 1 h at 37 °C and on a rocker. Cells in the circulatory perfusion compartment were collected by extracting media from the tubing, along with two washes of the tubing and circulatory compartment with FACS buffer. All cells were spun down, filtered using a Flowmi Cell Strainers (Millipore Sigma, BAH136800040), and re‐suspended in a 96‐well V‐Bottom plate (Corning, 3894). Cells were then blocked using FcR Blocking Solution (Milteyni, 130‐059‐901) for 20 min. Cells were subsequently incubated with either^[^ ^1^ ^]^ BV421‐CD45, APC‐CD34, BV605‐CD38, BUV395‐CD90, PE‐CD45RA, or.^[^ ^2^ ^]^ FITC‐CD45, BV421‐CD11b, BV605‐CD14, APC‐CD15, PE‐CD16, BUV395‐CD11c for 45 min on ice, stained with PI (ThermoFisher, P1304MP) for 15 min, washed, and analyzed on a Bio‐Rad ZE5 flow cytometer in 150 µL of FACS buffer. FlowJo (BD Biosciences) was used for the analysis of gating, cell populations, and median fluorescent intensity of different cell‐surface markers.

### Histological Analysis

All tissues (eBM, eCT, eLiv) were fixed in 4% paraformaldehyde overnight at 4 °C and washed three times with PBS. eBM tissues were subsequently decalcified using Osteosoft (Millipore Sigma, 1.01728) overnight at RT. eBM and eLiv tissues were paraffin‐embedded, sectioned at 5–6 µm, and stained for hematoxylin and eosin (H&E), Masson's Trichrome, and Picrosirius Red at Columbia's HICCC Histology and Pathology Core Facility. Paraffin sections were de‐paraffinized by sequential re‐hydration from CitriSolv and 100% ethanol to 100% distilled water. Tissue sections underwent antigen retrieval using 10 mm sodium citrate buffer (tri‐sodium citrate, distilled water, 1 m hydrochloric acid, and tween‐20), as previously reported.^[^ ^19^ ^]^ Sections were washed and permeabilized with 0.25% Triton‐X, blocked in 10% serum, and stained with tissue‐specific primary antibodies. eBM tissues were stained with CD45 (Mouse, AB1430). eLiv tissues were stained with Vimentin (Abcam, ab24525), CK18 (ReVMaB, 31‐1162‐00), anti‐cytochrome P450 CYP3A4 (Sigma, AB1254), COL1A1 (R&D Systems, MAB6220‐100), and ProLong Diamond Antifade Mountant with DAPI (ThermoFisher, P36962). Tissues were imaged using a Zeiss wide‐field optical microscope or scanning laser confocal microscope (A1 confocal system with Eclipse Ti inverted microscope, Nikon Instruments).

Whole‐mount cardiac tissues were fixed and permeabilized in ice‐cold methanol for 10 min, washed three times in PBS, and then blocked for 1 h at room temperature in PBS with 2% fetal bovine serum (FBS). After blocking, the tissues were incubated with primary mouse anti‐α‐actinin (sarcomeric) antibody (Sigma–Aldrich, A7811) as well as vimentin (Abcam, ab24525), washed three times and incubated for 1 h with secondary antibodies (Millipore Sigma, AP194C; Thermo Fischer, A‐21206; Thermo Fischer, A‐31571). For nuclei detection, the tissues were washed and incubated with NucBlue (Thermo Fisher, R37606). Whole tissues were placed in incubation chambers (Grace bio‐labs, 645501) and mounted with ProLong Diamond antifade mountant (Invitrogen, 36961). Samples were visualized using a scanning laser confocal microscope (A1 confocal system with Eclipse Ti inverted microscope, Nikon Instruments).

### Cytokine Assays

The supernatant was collected throughout the duration of the experiment and stored at −80 °C for <2 months prior to thawing and analyses. Supernatants from each tissue chamber and the circulatory compartment were spun down to remove cellular debris prior to each assay. For inflammatory cytokine analyses, the LegendPlex Human Inflammation Panel 1 (BioLegend, 740809) was used, according to the manufacturer's instructions. Human cardiac troponin T (Abcam, ab223860), Albumin (Bethyl, E88‐129), and Urea Nitrogen (Thermo Fisher, SB‐0580‐250) were measured according to the manufacturer's instructions.

### Bulk RNA Sequencing

RNA was isolated from circulating cells using a Qiagen RNeasy Micro Kit (Qiagen, 74004), according to the manufacturer's instructions, and analyzed for quality and quantity using an Agilent bioanalyzer, Qubit 2.0 at Columbia's Molecular Pathology core. For RNA sequencing, a SMART‐Seq v4 Ultra Low Input RNA Kit for Sequencing (TaKaRa) was used for cDNA amplification according to the manufacturer's instructions. Through Columbia's Genome Center, the library was prepared using Nextera XT DNA Library Preparation kit (Illumina) with 100–150 pg starting material, then followed the manufacturer's instructions. Libraries were sequenced to a targeted depth of ≈40 m 100 bp paired‐end reads on a NovaSeq 6000. RTA (Illumina) was used for base calling and bcl2fastq2 (version 2.19) for converting BCL to fastq format, coupled with adaptor trimming. A pseudoalignment was performed to a kallisto index created from transcriptomes (Human: GRCh38, kallisto 0.44.0). Data was processed first with DESeq2 for differential expression analysis.^[^ ^58^ ^]^ Gene ontology pathway analysis was performed using gProfiler and Revigo together to process data for Figures 5 and 6.

### Statistics

All data were analyzed by one‐ or two‐way ANOVA (as noted in figure captions) with outlier analysis and plotted by GraphPad Prism 9. Statistical significance was established at *p* < 0.05. For sequencing data analysis, statistical significance was established with FDR ≤ 0.05 and *p* ≤ 0.001. For pathway analysis, differentially expressed genes were considered as significant for log_2_FC ≥ 0.6.

## Conflict of Interest

The authors declare no conflict of interest.

## Supporting information

Supporting Information

## Data Availability

The data that support the findings of this study are available from the corresponding author upon reasonable request.
