# Supplementary material for: Modeling the Effects of Protracted Cosmic Radiation in a Human Organ‐on‐Chip Platform
Source: Adv Sci (Weinh). 2024 Jul 4;11(42):2401415. doi: 10.1002/advs.202401415 (PMC11558103; doi:10.1002/advs.202401415)
Supplement: Supplementary file 1 — Supporting Information [file ADVS-11-2401415-s001.pdf]

ADVANCED SCIENCE
Open Access

Supporting Information

for *Adv. Sci.*, DOI 10.1002/advs.202401415

Modeling the Effects of Protracted Cosmic Radiation in a Human Organ-on-Chip Platform

*Daniel Naveed Tavakol, Trevor R. Nash, Youngbin Kim, Pamela L. Graney, Martin Liberman, Sharon Fleischer, Roberta I. Lock, Aaron O'Donnell, Leah Andrews, Derek Ning, Keith Yeager, Andrew Harken, Naresh Deoli, Sally A. Amundson, Guy Garty, Kam W. Leong, David J. Brenner and Gordana Vunjak-Novakovic**

Supplementary Files

Modeling the effects of protracted cosmic radiation in a human organ-on-chip platform

Daniel Naveed Tavakol, Trevor R. Nash, Youngbin Kim, Pamela L. Graney, Martin Liberman, Sharon Fleischer, Roberta I. Lock, Aaron O'Donnell, Leah Andrews, Derek Ning, Keith Yeager, Andrew Harken, Naresh Deoli, Sally Amundson, Guy Garty, Kam W. Leong, David J. Brenner and Gordana Vunjak-Novakovic

Figure S1. Overview of neutron radiation facility and reactor irradiation set up
Figure S2. Schematic overview
Figure S3. Characterization of engineered human tissue models prior to integration
Figure S4. Example flow cytometric gating
Figure S5. Additional flow cytometric data of eBM compartment in suspension and adherent fractions at 2 weeks post-radiation
Figure S6. Integration of electrical stimulation into millipillar-designed cardiac muscle tissues
Figure S7. CIBERSORT predicted identification of blood populations in bulk RNA sequencing of circulating immune cells
Figure S8. Top genes in circulating, BM-derived immune cells characterized at 2 weeks post-radiation, via bulk RNA sequencing

Table S1. Top 40 significant DEGs in protracted versus acute circulating immune cells at the 2 week timepoint.
Table S2. Top 40 significant DEGs in protracted versus control circulating immune cells at the 2 week timepoint.
Table S3. Top 40 significant DEGs in acute versus control circulating immune cells at the 2 week timepoint.

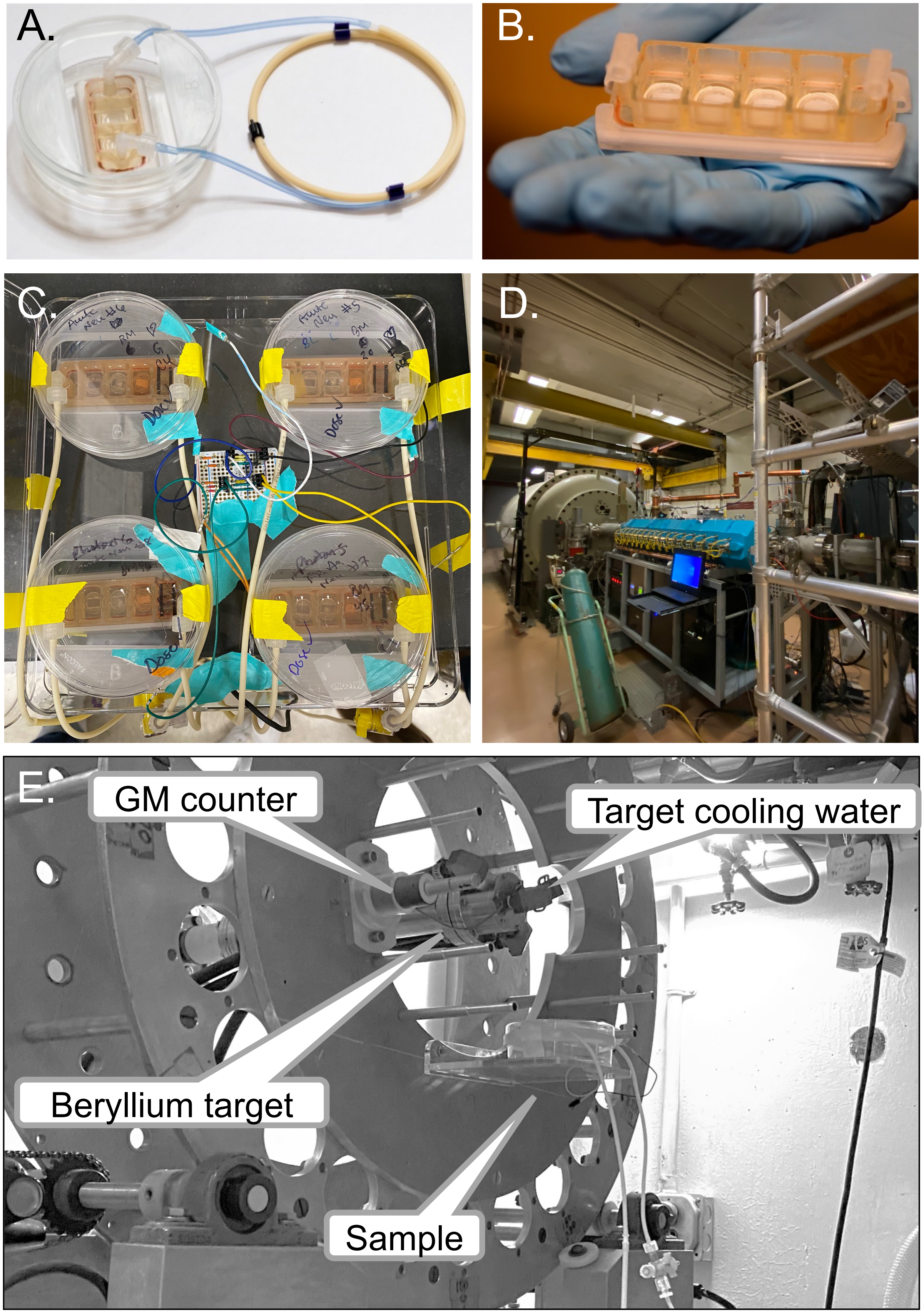

Figure S1. Overview of neutron radiation facility and reactor irradiation set up. (A, B) Example images of multi-organ reactor setup, as described in *(20)*. (C) Incubator storage set up of reactors connected to electrical stimulation Arduino. (D) Neutron accelerator with (E) zoomed in image of target of the beam and sample placement.

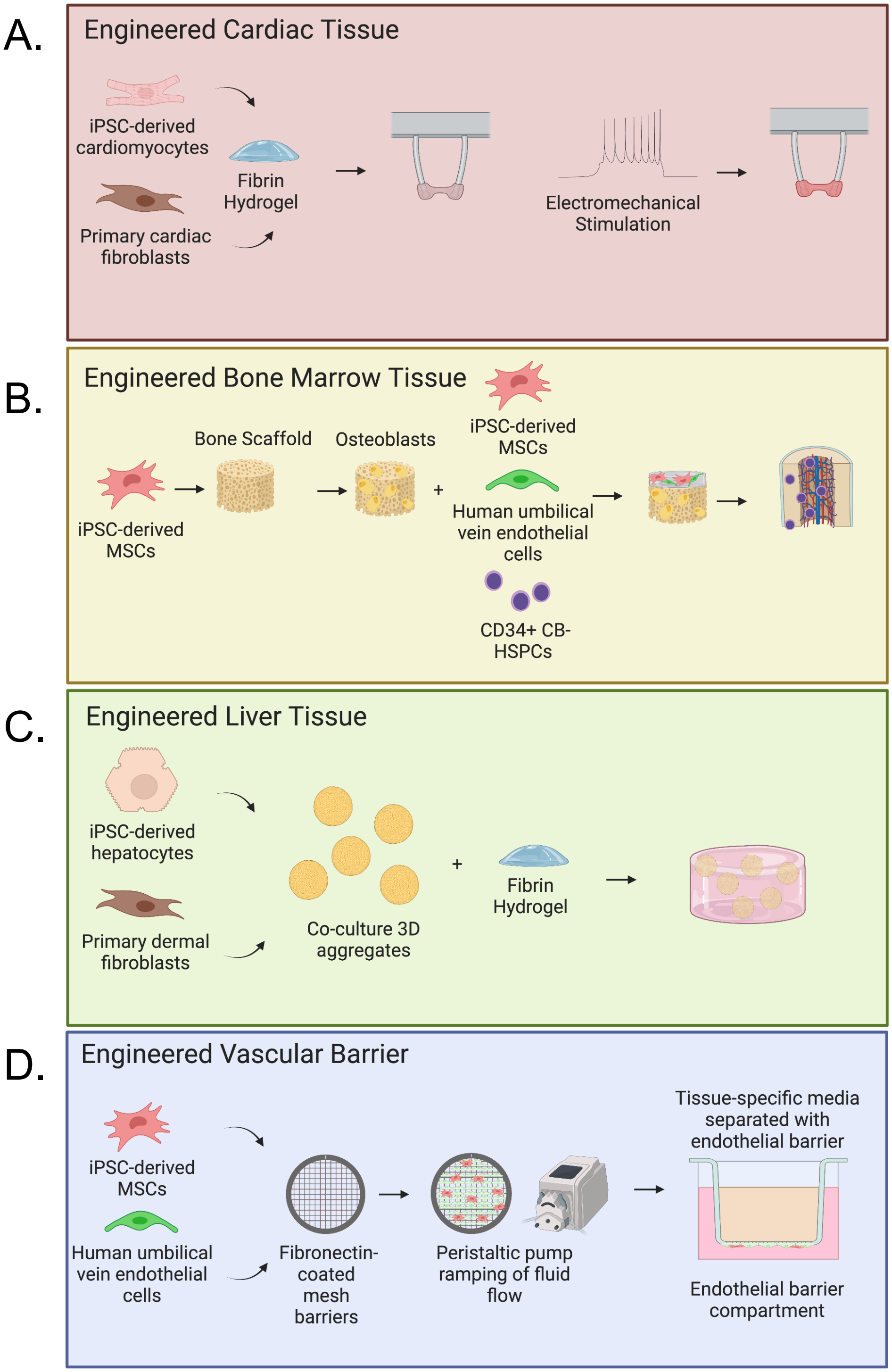

Figure S2. Schematic overview of individual tissue model fabrication prior to integration in multi-organ platform (see **Methods** for more details). (A) eCTs were formed by a combination of cardiomyocytes and fibroblasts and matured over a period of 4 weeks by electrical and metabolic conditioning. (B) eBM tissues were formed by adding MSCs to bone scaffolds, matured into osteoblasts, and further seeded with additional MSCs, endothelial cells, and HSPCs. (C) eLivs were formed by aggregating hepatocytes and fibroblasts into spheroids and culturing these spheroids within a 3D fibrin hydrogel. (D) Vascular barriers were formed by adding MSCs and endothelial cells onto custom fibronectin-coated mesh barriers prior to incremental ramping of flow prior to tissue integration.

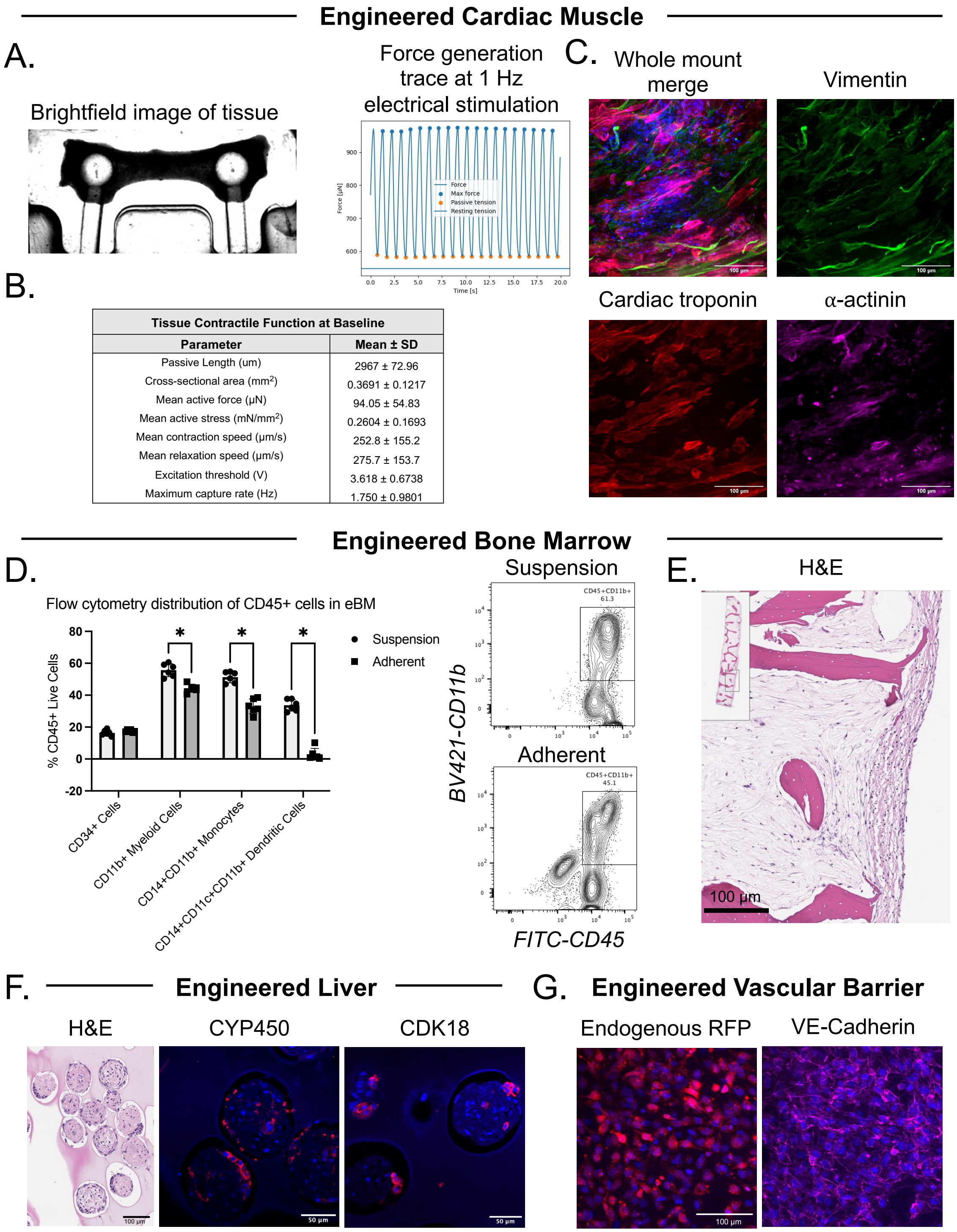

Tissue Contractile Function at Baseline	
**Parameter**	**Mean ± SD**
Passive Length (um)	2967 ± 72.96
Cross-sectional area (mm^2)	0.3691 ± 0.1217
Mean active force (μN)	94.05 ± 54.83
Mean active stress (mN/mm^2)	0.2604 ± 0.1693
Mean contraction speed (μm/s)	252.8 ± 155.2
Mean relaxation speed (μm/s)	275.7 ± 153.7
Excitation threshold (V)	3.618 ± 0.6738
Maximum capture rate (Hz)	1.750 ± 0.9801

Figure S3. Characterization of engineered human tissues prior to integration. Engineered cardiac tissues (eCT): (A) Example brightfield image of a eCT with representative force trace at 1 Hz of electrical stimulation. (B) Baseline functional data and (C) whole mount image of eCTs after maturation. Engineered bone marrow (eBM): (D) Flow cytometry characterization of hematopoietic cell types in suspension and adherent fractions of eBMs after 12 days of culture, with representative plots of both tissue fractions. $p < 0.05$ using multiple t-tests. (E) H&E staining of eBMs prior to integration. Engineered Liver (eLiv): (F) Representative staining of liver aggregates within fibrin hydrogels via H&E and immunostaining for CYP450 and CDK18. Engineered vascular barrier: (G) Representative staining of a barrier fabricated with endogenous RFP HUVECs and immunostaining for VE-Cadherin.

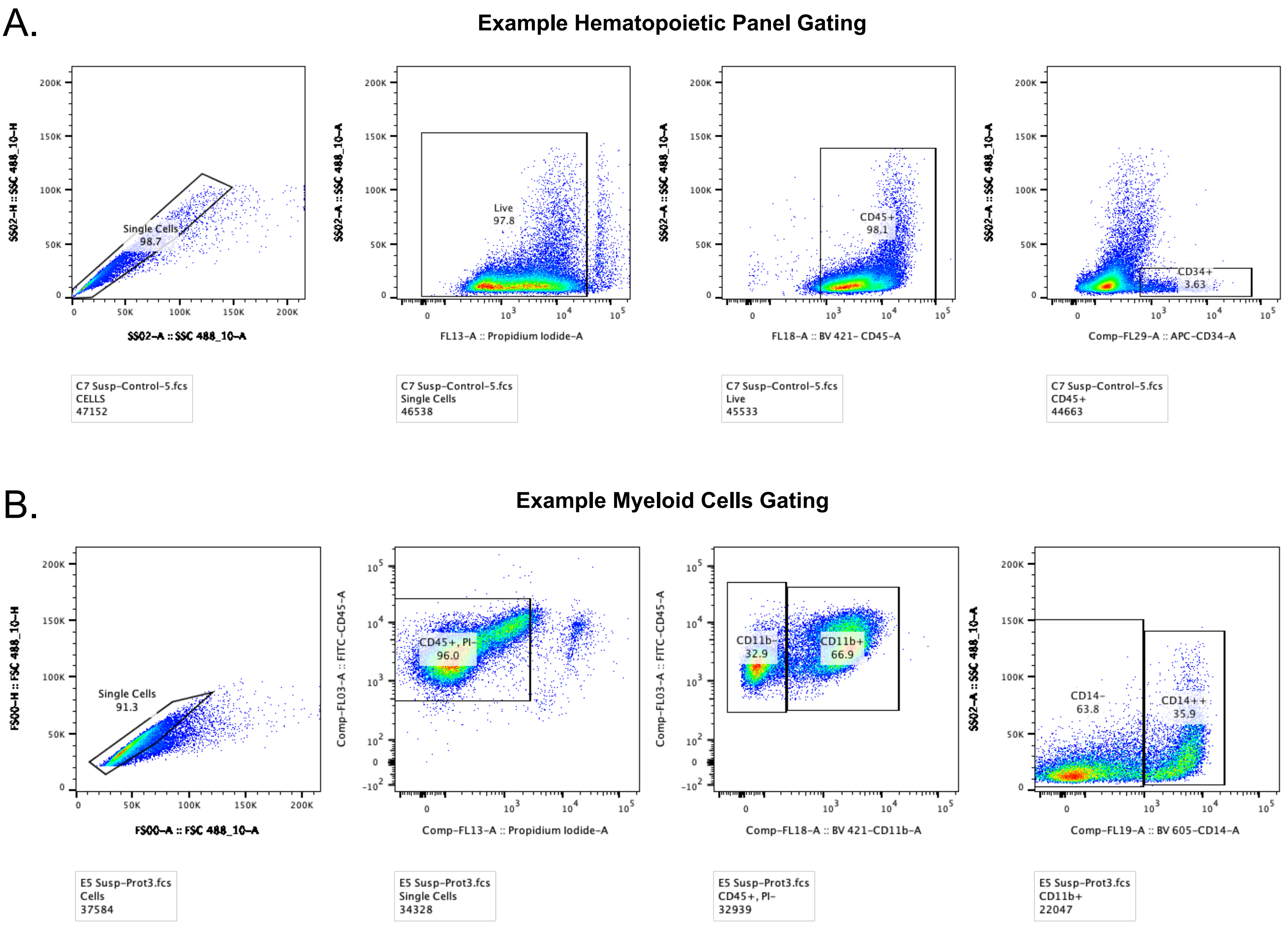

Figure S4. Example flow cytometric gating for isolated blood cells from eBM cultures to assess hematopoietic progenitor s (A) and myeloid differentiation (B).

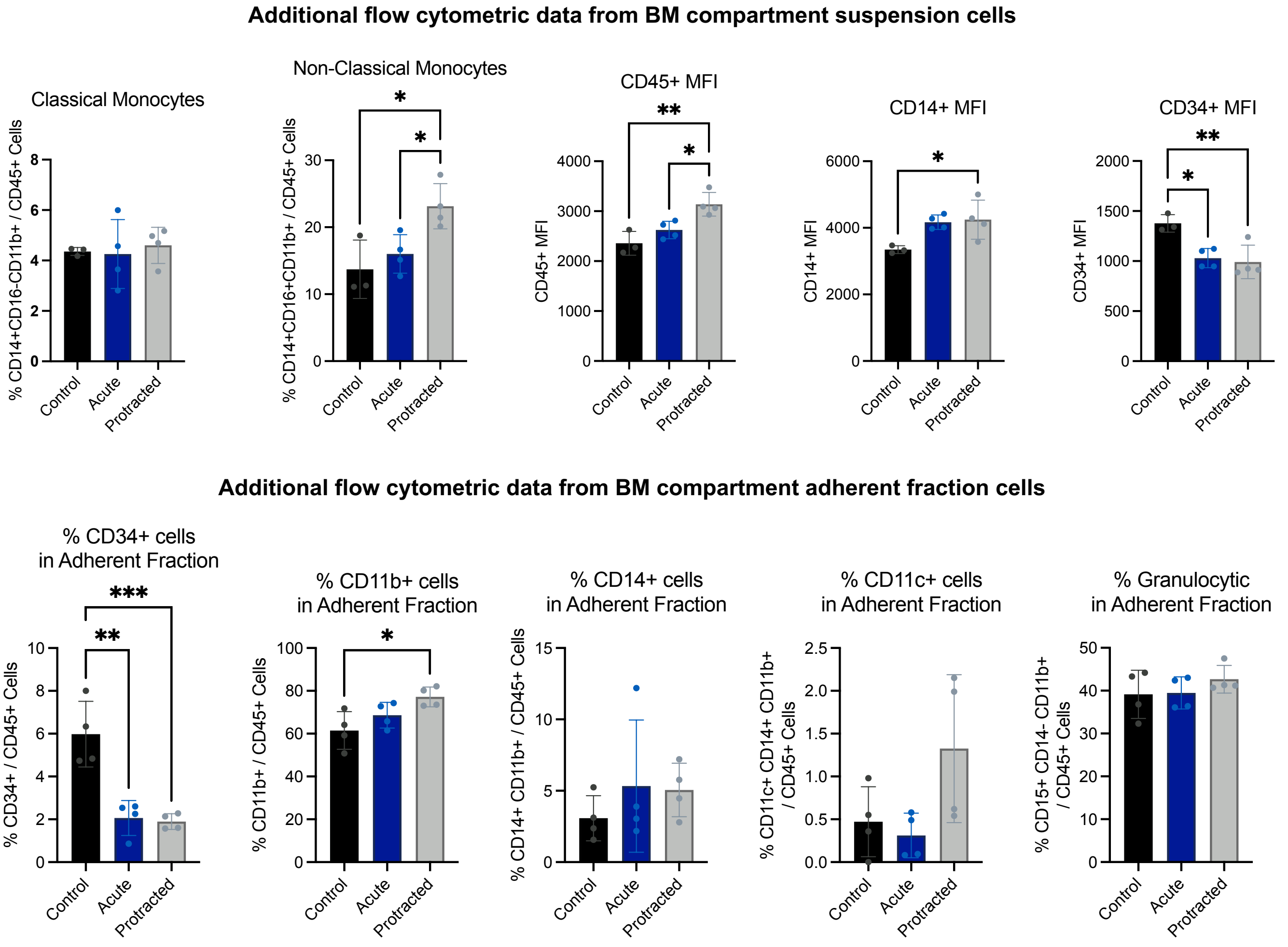

Figure S5. Additional flow cytometric data for suspension and adherent cell fractions in eBM at 2 weeks post-radiation. *p-value < 0.05; **p-value < 0.01; ***p-value < 0.005 with One-Way ANOVA.

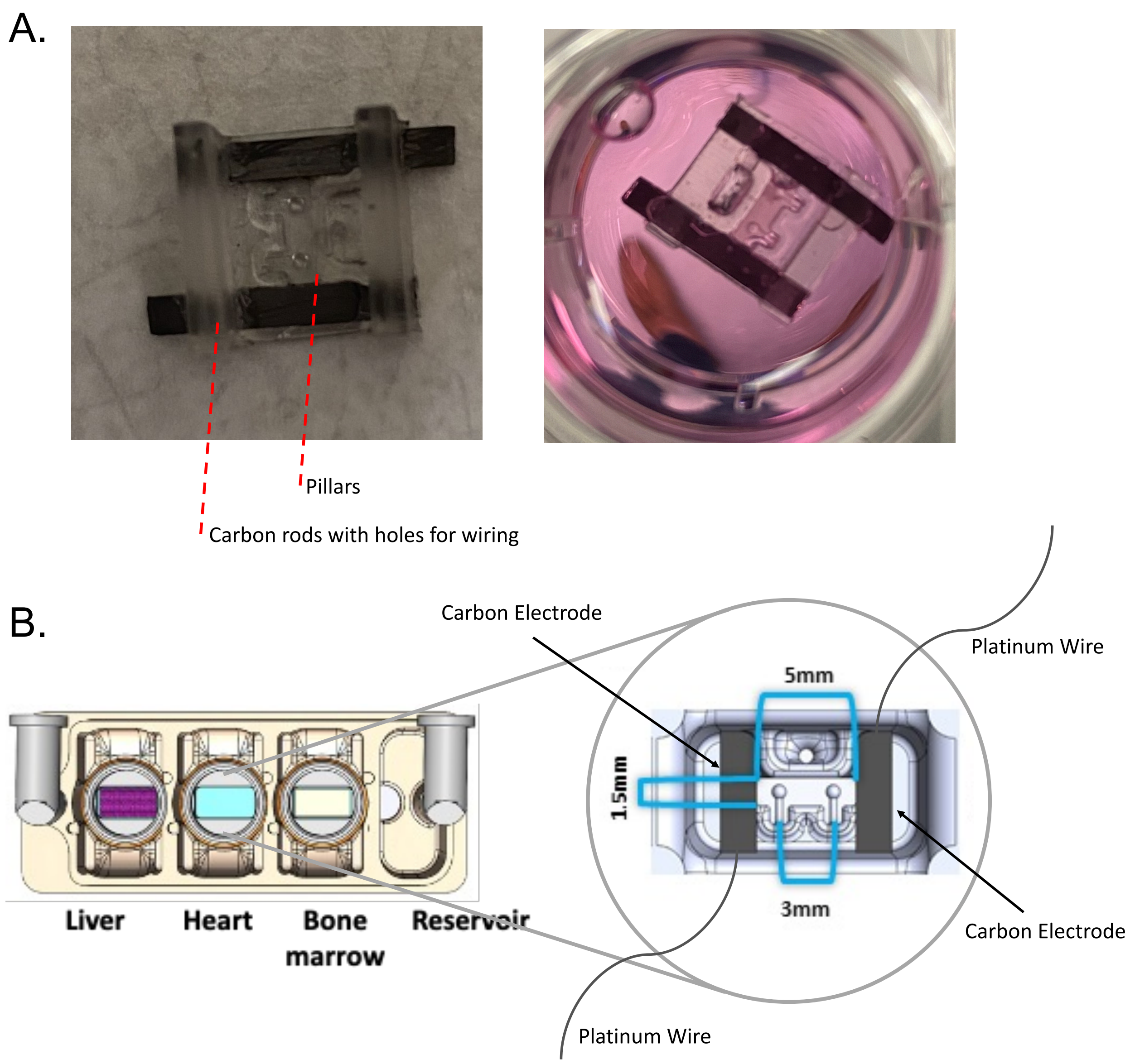

Figure S6. Integration of electrical stimulation into cardiac muscle tissue culture. (A) Image of cardiac tissue pillars between PDMS-encapsulated carbon electrodes. (B) Integration of cardiac tissues into the multi-organ platform to allow for platinum wires to extend outwards and connect to central electrical stimulation.

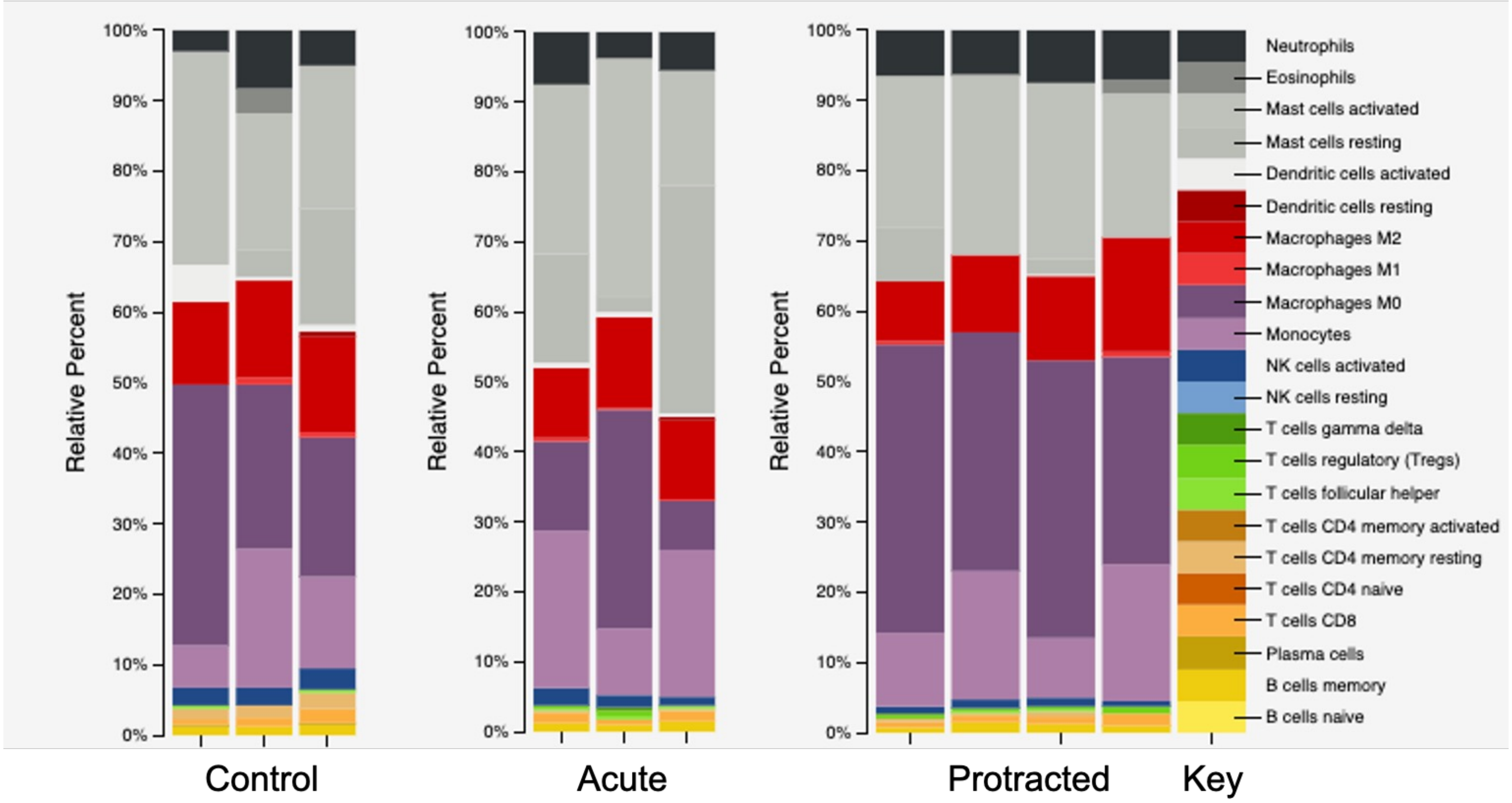

Figure S7. CIBERSORT predicted Identification of blood populations by bulk RNA sequencing of circulating immune cells. Data are shown at 2 weeks post-radiation.

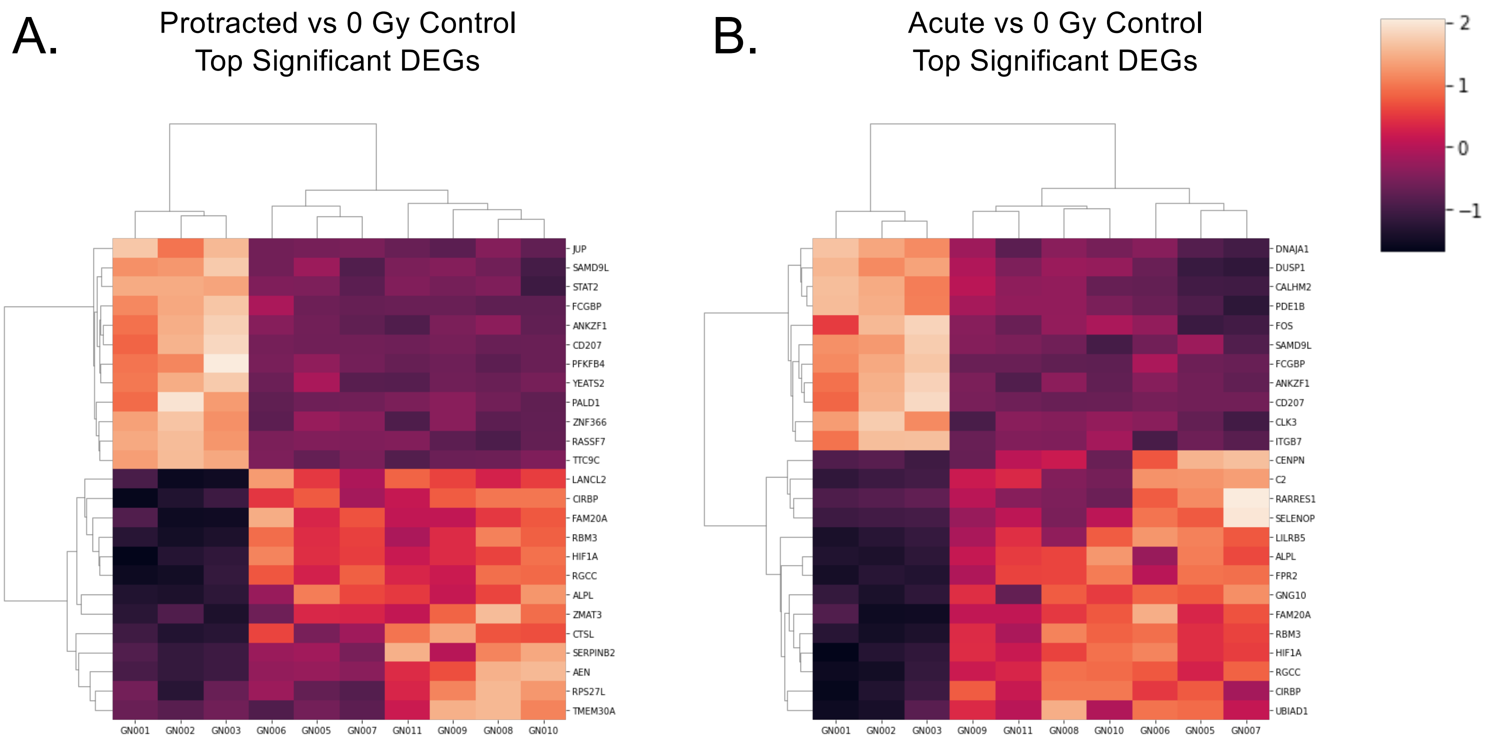

Figure S8. Top differentially expressed genes in circulating, eBM-derived immune cells. Data are shown **at 2 weeks post-radiation, via bulk RNA sequencing.** Comparisons of (A) protracted and (B) acute circulating immune cells relatively to controls.

Positive FC Protracted versus Acute			Negative FC Protracted versus Acute		
**Gene**	**FC**	**Padj**	**Gene**	**FC**	**Padj**
**NPIPA7**	4.614514	0.043072	**CAND2**	-5.521163	0.006488
**RIMBP3B**	4.404554	0.044539	**FAM178B**	-4.641352	0.006488
**LINC00475**	3.673574	0.025925	**EPB42**	-4.618839	0.027119
**ACSBG2**	3.616974	0.040604	**PKLR**	-4.525549	0.012477
**RHOXF1P1**	2.56931	0.042325	**LTA**	-4.016099	0.028694
**UNC13A**	2.377161	0.020926	**AHSP**	-3.800136	0.012798
**SPINK1**	2.298593	0.011199	**CTSE**	-3.649453	0.040324
**DPYSL3**	2.170625	0.036043	**NOX3**	-3.647402	0.027122
**IFNLR1**	1.843574	0.009123	**SHCBP1L**	-3.519487	0.01918
**SLC7A11**	1.811136	0.014535	**FSTL4**	-3.293815	0.04965
**COL8A2**	1.652558	0.015789	**CHRNA1**	-3.209643	0.021093
**TRIM16L**	1.633258	0.019278	**COL9A1**	-3.138992	0.022948
**COL7A1**	1.615069	0.043668	**CYP4F12**	-3.010654	0.049988
**MSC**	1.608758	0.014909	**HBA2**	-3.004337	0.003487
**EGR2**	1.548553	0.039372	**MKRN3**	-2.993442	0.047019
**OSGIN1**	1.524273	0.015193	**ADAM22**	-2.926243	0.036113
**IL3RA**	1.509556	0.007355	**CLEC9A**	-2.855714	0.032859
**MT1X**	1.500641	0.042563	**GYG2**	-2.817025	0.024886
**FAM187A**	1.496241	0.012798	**DNASE1L3**	-2.79646	0.037561
**TM4SF19**	1.482903	0.044098	**KRT72**	-2.793467	0.013639
**CCL7**	1.474414	0.044098	**CMA1**	-2.777168	0.011037
**CXCL3**	1.435666	0.020272	**CCDC136**	-2.66015	0.036113
**GRIN3B**	1.430224	0.034932	**SLC35D3**	-2.576885	0.016655
**HMOX1**	1.42586	0.036113	**HBB**	-2.504181	0.003487
**CSF1**	1.406834	0.035787	**HLA-DOB**	-2.449053	0.00707
**GPR68**	1.373206	0.006488	**FBXO43**	-2.401559	0.042804
**CXCL8**	1.365909	0.013757	**SLC16A9**	-2.278821	0.032067
**CDKN1A**	1.358329	0.003487	**RIBC2**	-2.259027	0.041472
**NQO1**	1.331996	0.022369	**GPC3**	-2.241114	0.042804
**MOB3B**	1.330882	0.013757	**PBK**	-2.162826	0.009123
**AKR1C7P**	1.307293	0.022464	**TCF15**	-2.142867	0.013757
**PHLDA3**	1.291866	0.025925	**TSPAN10**	-2.126606	0.014535
**ACTA2**	1.276834	0.006785	**UBASH3A**	-2.101044	0.040091
**AK8**	1.26546	0.039749	**ITLN1**	-2.098362	0.006785
**TMEM119**	1.249086	0.014163	**KLF1**	-2.040627	0.006488
**CXCL2**	1.236026	0.016751	**HLA-DQB2**	-2.034135	0.026962
**DAGLA**	1.198552	0.042804	**CXCR6**	-2.031369	0.047951
**TPST1**	1.190371	0.017375	**CA1**	-2.018891	0.042804
**SDC4**	1.174468	0.04135	**ANK3**	-1.948569	0.028538
**IL7R**	1.171728	0.013177	**VCAM1**	-1.92458	0.006488

Table S1. Top 40 significant differentially expressed genes in circulating immune cells 2 weeks following protracted versus acute radiation exposure.

Positive FC Protracted versus Control			Negative FC Protracted versus Control		
**Gene**	**FC**	**FDR**	**Gene**	**FC**	**FDR**
**CITED1**	4.685356	0.028105	**CD207**	-5.472017	0.000112
**EDA2R**	4.457223	0.009106	**CAND2**	-5.424021	0.001541
**WNT2**	4.169367	0.041517	**APLN**	-4.504483	0.012903
**MSX1**	4.098062	0.007964	**EIF3CL**	-4.243928	0.028499
**HBE1**	3.819239	0.013164	**GPR17**	-4.241629	0.013038
**PRR5-ARHGAP8**	3.686852	0.007703	**KLHL30**	-3.905117	0.003559
**C2orf16**	3.674192	0.025743	**PLA2G4B**	-3.812906	0.045812
**PRRX2**	3.647634	0.042205	**TRIM6-TRIM34**	-3.743578	0.012993
**APOBEC3H**	3.413957	0.002334	**CYP2J2**	-3.718933	0.02771
**GFRA2**	3.413029	0.047726	**FCGBP**	-3.695146	0.000112
**KLF14**	3.306748	0.028026	**OTOF**	-3.612167	0.040574
**RAI2**	3.121528	0.045001	**CDRT4**	-3.58794	0.023009
**LINC00475**	3.092751	0.007487	**CNNM1**	-3.547635	0.036473
**COL10A1**	3.08654	0.021969	**EFCC1**	-3.461575	0.024684
**ALPL**	3.027047	0.0003	**KISS1R**	-3.426755	0.027996
**DKK3**	3.024487	0.015446	**EPHA2**	-3.214571	0.012971
**MUCL1**	2.901124	0.009082	**IGHM**	-3.204509	0.000738
**SPATA18**	2.876559	0.006288	**IFI44L**	-3.075658	0.001786
**MFSD4BP1**	2.754646	0.042732	**KCNN1**	-3.058718	0.029772
**C3orf52**	2.711362	0.007045	**CRHBP**	-3.043976	0.032531
**PRRG3**	2.700231	0.046213	**LTA**	-2.935164	0.048662
**SERPINB2**	2.678406	0.000353	**CERS1**	-2.864754	0.01503
**UCN2**	2.67119	0.005503	**AMPH**	-2.850921	0.017964
**MT1L**	2.659409	0.03189	**MKRN3**	-2.819722	0.028437
**CDC42EP5**	2.651675	0.026005	**SDK2**	-2.791173	0.019229
**NTM**	2.638704	0.021846	**NCR2**	-2.75419	0.03189
**FOXF1**	2.625129	0.023409	**CDH1**	-2.744159	0.000549
**MYOZ1**	2.61325	0.036859	**LRRC43**	-2.743788	0.046045
**RND3**	2.504581	0.001461	**GCSAM**	-2.697785	0.03525
**FBLN5**	2.424076	0.008704	**INA**	-2.675728	0.025889
**STEAP1**	2.370028	0.012971	**CD69**	-2.639076	0.003483
**LRRC39**	2.355671	0.047867	**CD200**	-2.560102	0.016581
**TMEM26**	2.256388	0.004515	**ZNF132**	-2.452218	0.020614
**BZW1P2**	2.190395	0.010277	**ENOX1**	-2.40461	0.01916
**MIXL1**	2.182318	0.039559	**IFI44**	-2.332552	0.000851
**CXCL12**	2.172916	0.033706	**OAS2**	-2.284093	0.001986
**VWCE**	2.171697	0.008716	**MX1**	-2.247153	0.002199
**ERMN**	2.166917	0.038519	**KLRK1**	-2.23021	0.009518
**NIPAL4**	2.165792	0.030987	**MAS1LP1**	-2.144052	0.030359
**CTHRC1**	2.138146	0.040712	**IFIT1**	-2.122453	0.004189

Table S2. Top 40 differentially expressed genes in immune cells at 2 weeks protracted radiation exposure, relatively to controls.

Positive FC Acute versus Control			Negative FC Acute versus Control		
**Gene**	**FC**	**Padj**	**Gene**	**FC**	**Padj**
**IL13RA2**	5.710793	0.019485	**TRIM6-TRIM34**	-7.285115	0.002804
**FMN2**	5.285637	0.01346	**ZBED6**	-5.604547	0.027874
**MSX1**	5.112467	0.001486	**INA**	-4.93477	0.005491
**HSPB2**	4.522336	0.015793	**ARHGEF34P**	-4.732974	0.012271
**PKLR**	4.473244	0.002223	**PHACTR2P1**	-4.518694	0.011902
**BORCS7-ASMT**	4.45331	0.015762	**RIMBP3B**	-4.239537	0.016728
**DOK5**	4.40211	0.024985	**GPR17**	-4.227155	0.012002
**EPB42**	4.154111	0.008194	**CD207**	-4.114032	0.000221
**PRRX2**	4.135537	0.016096	**KISS1R**	-4.023474	0.01582
**ADAMTS5**	4.099037	0.046194	**GLP2R**	-3.957501	0.033087
**KRT2**	4.081801	0.017091	**EPHA2**	-3.782089	0.008704
**FIBIN**	4.063072	0.047729	**MYH16**	-3.606724	0.038644
**MRGPRF**	4.030804	0.026818	**OTOF**	-3.540403	0.033433
**DKK1**	4.000869	0.04487	**CD200**	-3.465003	0.007398
**DEFT1P**	3.994925	0.016855	**PRSS8**	-3.255942	0.00046
**EDA2R**	3.96397	0.011121	**CDRT4**	-3.207674	0.020801
**KIRREL1**	3.868877	0.032837	**ACSBG2**	-3.150058	0.024195
**FOXF1**	3.580153	0.002716	**THEMIS3P**	-3.110085	0.023781
**DPT**	3.565521	0.024968	**GALNT18**	-3.105048	0.008924
**SVEP1**	3.546702	0.024512	**MT1H**	-3.10132	0.015856
**CPB1**	3.480273	0.03628	**EGF**	-3.07186	0.029723
**PCDH18**	3.466236	0.017487	**OR2A7**	-3.031797	0.03729
**TAF7L**	3.449176	0.013763	**HSPA1B**	-2.85953	0.000729
**HS3ST2**	3.395821	0.000373	**FCGBP**	-2.802889	0.000281
**RPE65**	3.360122	0.024358	**KCNJ11**	-2.746646	0.047031
**DCN**	3.334563	0.007002	**CD69**	-2.682295	0.00273
**FOLR2**	3.245092	0.00043	**CLDN1**	-2.673509	0.001788
**SELENOP**	3.240936	0.000118	**EFCC1**	-2.655461	0.041078
**TRNP1**	3.196321	0.035773	**TCP10L**	-2.649041	0.04085
**FLNC**	3.194838	0.02498	**NCR2**	-2.591245	0.033735
**C3orf52**	3.183512	0.00189	**CNTNAP3B**	-2.493779	0.025261
**RND3**	3.144122	0.000392	**CLIC3**	-2.486053	0.034482
**ITLN1**	3.130496	0.00057	**ZNF610**	-2.473991	0.008932
**MAG**	3.127702	0.01346	**DQX1**	-2.470788	0.008016
**IRX3**	3.097191	0.046081	**ZBTB26**	-2.458425	0.041385
**IL23R**	3.092902	0.040878	**COLGALT2**	-2.454776	0.004375
**MMP11**	3.085381	0.026933	**LPAR3**	-2.435732	0.000791
**CCN2**	3.052591	0.042709	**RIMBP3**	-2.430589	0.007329
**AHSP**	3.038246	0.003669	**RPL7AP64**	-2.376955	0.006179
**FAM131B**	3.030846	0.049465	**UNC13A**	-2.353278	0.005053

Table S3. Top 40 differentially expressed genes in circulating immune cells at 2 weeks acute radiation exposure relatively to control
